# Thalamus involvement in genetic frontotemporal dementia assessed using structural and diffusion MRI: a GENFI study

**DOI:** 10.1093/braincomms/fcaf420

**Published:** 2025-10-24

**Authors:** Sonja Soskic, Henry F J Tregidgo, Emily G Todd, Arabella Bouzigues, David M Cash, Lucy L Russell, David L Thomas, Ian B Malone, Phoebe H Foster, Eve Ferry-Bolder, John C van Swieten, Lize C Jiskoot, Harro Seelaar, Raquel Sanchez-Valle, Robert Laforce, Caroline Graff, Daniela Galimberti, Rik Vandenberghe, Alexandre de Mendonça, Pietro Tiraboschi, Isabel Santana, Alexander Gerhard, Johannes Levin, Benedetta Nacmias, Markus Otto, Maxime Bertoux, Thibaud Lebouvier, Simon Ducharme, Christopher R Butler, Isabelle Le Ber, Elizabeth C Finger, Maria Carmela Tartaglia, Mario Masellis, James B Rowe, Matthis Synofzik, Fermin Moreno, Barbara Borroni, Daniel C Alexander, Juan Eugenio Iglesias, Jonathan D Rohrer, Martina Bocchetta, GENetic Frontotemporal dementia Initiative (GENFI), Rhian Convery, Sophie Goldsmith, Kiran Samra, Thomas Cope, Maura Malpetti, Antonella Alberici, Enrico Premi, Roberto Gasparotti, Emanuele Buratti, Valentina Cantoni, Andrea Arighi, Chiara Fenoglio, Vittoria Borracci, Maria Serpente, Tiziana Carandini, Emanuela Rotondo, Giacomina Rossi, Giorgio Giaccone, Giuseppe Di Fede, Paola Caroppo, Sara Prioni, Veronica Redaelli, David Tang-Wai, Ekaterina Rogaeva, Johanna Krüger, Miguel Castelo-Branco, Morris Freedman, Ron Keren, Sandra Black, Sara Mitchell, Christen Shoesmith, Robart Bartha, Rosa Rademakers, Jackie Poos, Janne M Papma, Lucia Giannini, Liset de Boer, Julie de Houwer, Rick van Minkelen, Yolande Pijnenburg, Camilla Ferrari, Cristina Polito, Gemma Lombardi, Valentina Bessi, Enrico Fainardi, Stefano Chiti, Mattias Nilsson, Henrik Viklund, Melissa Taheri Rydell, Vesna Jelic, Abbe Ullgren, Tobias Langheinrich, Albert Lladó, Anna Antonell, Jaume Olives, Mircea Balasa, Nuria Bargalló, Sergi Borrego-Ecija, Ana Verdelho, Carolina Maruta, Tiago Costa-Coelho, Gabriel Miltenberger, Frederico Simões do Couto, Alazne Gabilondo, Ioana Croitoru, Mikel Tainta, Myriam Barandiaran, Patricia Alves, Benjamin Bender, David Mengel, Lisa Graf, Annick Vogels, Mathieu Vandenbulcke, Philip Van Damme, Rose Bruffaerts, Koen Poesen, Pedro Rosa-Neto, Maxime Montembault, Raffaella Migliaccio, Ninon Burgos, Daisy Rinaldi, Catharina Prix, Elisabeth Wlasich, Olivia Wagemann, Sonja Schönecker, Alexander Maximilian Bernhardt, Anna Stockbauer, Jolina Lombardi, Sarah Anderl-Straub, Adeline Rollin, Gregory Kuchcinski, Vincent Deramecourt, João Durães, Marisa Lima, Maria João Leitão, Maria Rosario Almeida, Miguel Tábuas-Pereira, Sónia Afonso, João Lemos

**Affiliations:** Department of Medical Physics and Biomedical Engineering, UCL Hawkes Institute, University College London, London WC1V 6LJ, UK; Department of Medical Physics and Biomedical Engineering, UCL Hawkes Institute, University College London, London WC1V 6LJ, UK; Department of Neurodegenerative Disease, Dementia Research Centre, UCL Queen Square Institute of Neurology, London WC1N 3BG, UK; Department of Clinical Neurosciences and Cambridge University Hospitals NHS Trust, University of Cambridge, Cambridge CB2 1TN, UK; Department of Neurodegenerative Disease, Dementia Research Centre, UCL Queen Square Institute of Neurology, London WC1N 3BG, UK; Department of Neurodegenerative Disease, Dementia Research Centre, UCL Queen Square Institute of Neurology, London WC1N 3BG, UK; UK Dementia Research Institute at University College London, London WC1N 3BG, UK; Department of Neurodegenerative Disease, Dementia Research Centre, UCL Queen Square Institute of Neurology, London WC1N 3BG, UK; Neuroradiological Academic Unit, Department of Brain Repair and Rehabilitation, UCL Institute of Neurology, Queen Square, University College London, London WC1N 3BG, UK; Department of Neurodegenerative Disease, Dementia Research Centre, UCL Queen Square Institute of Neurology, London WC1N 3BG, UK; Department of Neurodegenerative Disease, Dementia Research Centre, UCL Queen Square Institute of Neurology, London WC1N 3BG, UK; Department of Neurodegenerative Disease, Dementia Research Centre, UCL Queen Square Institute of Neurology, London WC1N 3BG, UK; Department of Neurology, Erasmus Medical Centre, Rotterdam 2040 3000, The Netherlands; Department of Neurology, Erasmus Medical Centre, Rotterdam 2040 3000, The Netherlands; Department of Neurology, Erasmus Medical Centre, Rotterdam 2040 3000, The Netherlands; Alzheimer’s Disease and Other Cognitive Disorders Unit, Neurology Service, Hospital Clínic, Institut d’Investigacións Biomèdiques August Pi I Sunyer, University of Barcelona, Barcelona 08036, Spain; Clinique Interdisciplinaire de Mémoire, Département des Sciences Neurologiques, CHU de Québec, and Faculté de Médecine, Université Laval, Québec City, QC G1V 0A6, Canada; Department of Neurobiology, Care Sciences and Society, Center for Alzheimer Research, Division of Neurogeriatrics, Bioclinicum, Karolinska Institutet, Solna 17177, Sweden; Unit for Hereditary Dementias, Theme Inflammation and Aging, Karolinska University Hospital, Solna 17177, Sweden; Fondazione Ca’ Granda, IRCCS Ospedale Policlinico, Milan 20122, Italy; Centro Dino Ferrari, University of Milan, Milan 20122, Italy; Laboratory for Cognitive Neurology, Department of Neurosciences, KU Leuven, Leuven 3000, Belgium; Neurology Service, University Hospitals Leuven, Leuven 3000, Belgium; Leuven Brain Institute, KU Leuven, Leuven 3000, Belgium; Faculty of Medicine, University of Lisbon, Lisbon 1649-004, Portugal; Fondazione IRCCS Istituto Neurologico Carlo Besta, Milano 20133, Italy; Neurology Service, Faculty of Medicine, University Hospital of Coimbra (HUC), University of Coimbra, Coimbra 3000-214, Portugal; Center for Neuroscience and Cell Biology, Faculty of Medicine, University of Coimbra, Coimbra 3000-214, Portugal; Division of Psychology Communication and Human Neuroscience, Wolfson Molecular Imaging Centre, University of Manchester, Manchester M13 9GB, UK; Department of Nuclear Medicine, Center for Translational Neuro- and Behavioral Sciences, University Medicine Essen, Essen 47057, Germany; Department of Geriatric Medicine, Klinikum Hochsauerland, Arnsberg 59759, Germany; Department of Neurology, Ludwig-Maximilians Universität München, Munich 80539, Germany; German Center for Neurodegenerative Diseases (DZNE), Munich 81377, Germany; Munich Cluster of Systems Neurology (SyNergy), Munich 81377, Germany; Department of Neuroscience, Psychology, Drug Research and Child Health, University of Florence, Florence 50139, Italy; IRCCS Fondazione Don Carlo Gnocchi, Florence 50143, Italy; Department of Neurology, University of Ulm, Ulm 89081, Germany; Lille Neuroscience & Cognition U1172, University of Lille, Inserm, CHU Lille, Lille 59000, France; Lille Neuroscience & Cognition U1172, University of Lille, Inserm, CHU Lille, Lille 59000, France; Department of Psychiatry, Douglas Mental Health University Institute, McGill University, Montreal, Québec H3H 2R9, Canada; McConnell Brain Imaging Centre, Montreal Neurological Institute, McGill University, Montreal, Québec H3H 2R9, Canada; Nuffield Department of Clinical Neurosciences, Medical Sciences Division, University of Oxford, Oxford OX1 4BH, UK; Department of Brain Sciences, Imperial College London, London SW7 2BX, UK; Département de Neurologie, AP-HP—Hôpital Pitié-Salpêtrière, Centre de référence des démences rares ou précoces, IM2A, Paris 5783, France; Department of Clinical Neurological Sciences, University of Western Ontario, London, ON N6A 5A5, Canada; Tanz Centre for Research in Neurodegenerative Diseases, University of Toronto, Toronto, ON M4N 3M5, Canada; Sunnybrook Health Sciences Centre, Sunnybrook Research Institute, University of Toronto, Toronto M4N 3M5, Canada; Department of Clinical Neurosciences and Cambridge University Hospitals NHS Trust, University of Cambridge, Cambridge CB2 1TN, UK; Department of Neurodegenerative Diseases, Hertie-Institute for Clinical Brain Research and Center of Neurology, University of Tübingen, Tübingen 72074, Germany; Center for Neurodegenerative Diseases (DZNE), Tübingen 72076, Germany; Cognitive Disorders Unit, Department of Neurology, Hospital Universitario Donostia, San Sebastian 20014, Spain; Neurosciences Area, Group of Neurodegenerative Diseases, Biogipuzkoa Health Research Institute, San Sebastian 20014, Spain; Center for Biomedical Research in Neurodegenerative Disease (CIBERNED), Carlos III Health Institute, Madrid 28029, Spain; Neurology Unit, Department of Clinical and Experimental Sciences, University of Brescia, Brescia 25123, Italy; Molecular Markers Laboratory, IRCCS Istituto Centro San Giovanni di Dio Fatebenefratelli, Brescia 25125, Italy; Department of Computer Science, UCL Hawkes Institute, University College London, London WC1V 6LJ, UK; Department of Medical Physics and Biomedical Engineering, UCL Hawkes Institute, University College London, London WC1V 6LJ, UK; Martinos Center for Biomedical Imaging, Massachusetts General Hospital and Harvard Medical School, Boston, MA 02114, USA; Computer Science and Artificial Intelligence Laboratory, Massachusetts Institute of Technology, Cambridge, MA 02139, USA; Department of Neurodegenerative Disease, Dementia Research Centre, UCL Queen Square Institute of Neurology, London WC1N 3BG, UK; Department of Neurodegenerative Disease, Dementia Research Centre, UCL Queen Square Institute of Neurology, London WC1N 3BG, UK; Centre for Cognitive and Clinical Neuroscience, Division of Psychology, Department of Life Sciences, College of Health, Medicine and Life Sciences, Brunel University London, London UB8 3PH, UK

**Keywords:** frontotemporal dementia, genetics, thalamus, MRI, diffusion tensor imaging

## Abstract

Thalamic subregions are commonly, but variably, affected by different forms of frontotemporal dementia. We aimed to better characterize thalamic subregional involvement in genetic frontotemporal dementia with a recently published thalamus segmentation tool that utilizes structural and diffusion MRI, offering additional assessment of mean diffusivity and a more fine-grained analysis of the pulvinar specifically compared to previous studies. Using this tool, we performed thalamus segmentations in MRI scans from *C9orf72*, *GRN* and *MAPT* mutation carriers and mutation non-carriers with suitable 3-Tesla MRI cross-sectional data from the GENetic Frontotemporal dementia Initiative. Mutation carriers were divided according to their genetic group and Clinical Dementia Rating® Dementia Staging Instrument plus National Alzheimer’s Coordinating Center Behaviour and Language Domains global score (0 or 0.5: presymptomatic/prodromal stage, 1 or higher: symptomatic stage). Following stringent quality control and harmonization across sites and scanners, we compared volumes and mean diffusivity values of thalamic subregions in *C9orf72* (47 presymptomatic, 10 symptomatic), *GRN* (57 presymptomatic, 11 symptomatic) and *MAPT* (31 presymptomatic, 12 symptomatic) mutation carriers to those in 109 mutation non-carriers with analyses of covariance including age and sex (and total intracranial volume for volumetric comparisons) as covariates. Presymptomatic *C9orf72* expansion carriers showed smaller volumes (3–8% difference from non-carriers) and higher mean diffusivity (2–5% difference from non-carriers) for several thalamic subregions, including all pulvinar subdivisions. We found subtly larger volumes of the ventral anterior subregion and the non-medial pulvinar (3% difference from non-carriers for both) in presymptomatic *GRN* mutation carriers, and of the anteroventral subregion (5% difference from non-carriers) in presymptomatic *MAPT* mutation carriers. Symptomatic mutation carriers in all three genetic groups showed significantly smaller volumes and widespread higher mean diffusivity of thalamic subregions compared with non-carriers, which were overall most prominent in subregions involved in associative and limbic functions (the midline, medial pulvinar, anteroventral, mediodorsal, laterodorsal and lateral posterior subregions). Notably smaller volume (12–23% difference from non-carriers) and higher mean diffusivity (16–23% difference from non-carriers) of the most medial part of the medial pulvinar was a shared feature across the three genetic groups at the symptomatic stage. Overall, our study confirms that thalamic subregions are affected in genetic frontotemporal dementia and identifies prominent involvement of the most medial part of the medial pulvinar as a potential unifying feature in the variable pattern of thalamic subregional involvement across the main genetic groups.

## Teaser

Soskic *et al*. investigated the involvement of thalamic subregions in genetic frontotemporal dementia using structural and diffusion MRI data. The authors found thalamic subregions to be affected at presymptomatic and symptomatic stages, with prominent medial pulvinar involvement being a shared feature amongst the main genetic groups at the symptomatic stage.

## Graphical Abstract

**Graphical Abstract:**
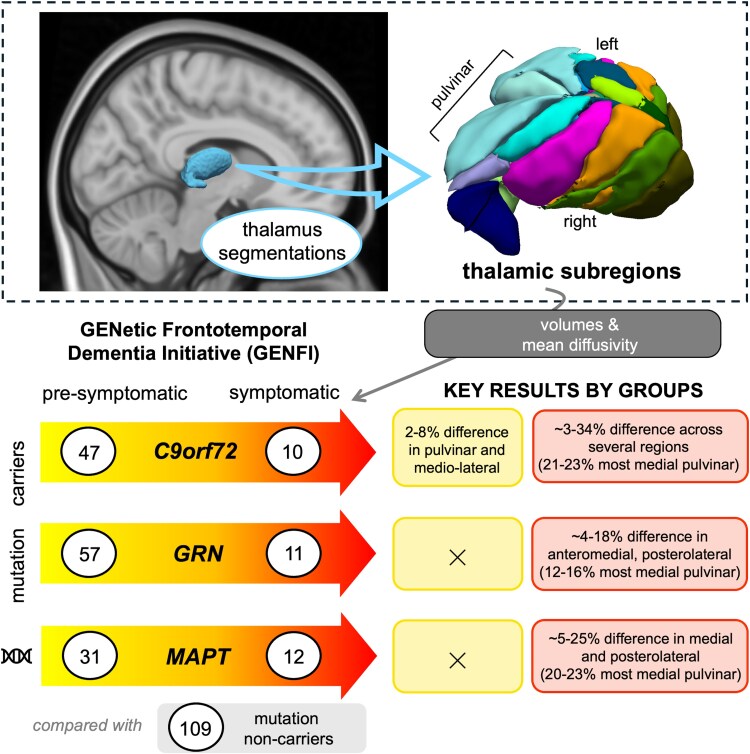

## Introduction

Genetic frontotemporal dementia (FTD) is a spectrum of heterogeneous neurodegenerative disorders, most commonly caused by an autosomal dominant mutation in the chromosome 9 open reading frame 72 (*C9orf72*), progranulin (*GRN*) or microtubule-associated protein tau (*MAPT*) genes, with variable age of disease onset.^1^ The pathogenic *C9orf72* and *GRN* mutations lead to intracellular accumulation of the transactive response DNA binding protein 43 kDa (TDP-43), whilst pathogenic *MAPT* mutations lead to intracellular aggregates of tau.^2^ Despite the known association between the affected gene and the likely type of pathology, the clinical and neuroanatomical features between individuals carrying mutations in the same gene are extremely heterogeneous. Classically, the frontal and temporal lobes of the brain are affected, leading to progressive changes in behaviour and personality (behavioural variant FTD, bvFTD), difficulties with speech and language (primary progressive aphasia, PPA) or motor symptoms (progressive supranuclear palsy—PSP, corticobasal syndrome—CBS, or amyotrophic lateral sclerosis—ALS).^3^

The GENetic Frontotemporal dementia Initiative (GENFI) is an international multicentre study established in 2012 that has been following FTD mutation carriers and their first-degree relatives annually with clinical, cognitive, imaging, genetic and fluid biomarker examinations.^4^ The longitudinal follow-up of individuals who are known mutation carriers offers an invaluable opportunity to investigate when and where brain pathology first develops in FTD prior to overt symptom onset. Determining this would help in better understanding the evolution of the disease and in establishing biomarkers that can track disease progression, which are needed for clinical trial design.

One particular region of the brain with good potential to provide imaging biomarkers for FTD is the thalamus, a highly organized subcortical structure connected with all areas of the cortex and with other subcortical regions.^5^ It is composed of numerous nuclei, many of which have distinct patterns of connections with other brain areas. As such, measures of thalamic nuclei volumes and their microstructural properties could capture not only primary localized pathology, but also pathology occurring elsewhere in the brain that is reflected in secondary thalamic changes.^6^

Imaging studies have shown *in vivo* that thalamic subregional volumes are reduced across the clinical, pathological and genetic forms of FTD at the symptomatic stage with variable atrophy patterns.^7,8^ In addition, thalamic subregional atrophy is already present at an early presymptomatic stage in *C9orf72* repeat expansion carriers, most prominently in the pulvinar region.^7,9,10^

These findings are based on a thalamus parcellation method that uses participants’ structural T1-weighted (T1w) magnetic resonance imaging (MRI) data.^11^ A recently developed thalamus parcellation tool jointly incorporates structural and diffusion MRI data^12^ to improve parcellation accuracy by utilising the different contrasts these two MRI modalities offer. More specifically, this tool uses fractional anisotropy (FA; a normalized measure of how strongly directional diffusion is in a voxel) and the principal eigenvector (the orientation of maximal diffusion) from diffusion tensor imaging (DTI), together with intensity distributions from structural MRI and a histology-derived probabilistic atlas within a Bayesian framework. In addition to improved parcellation, the DTI contrast allows the medial pulvinar, a region of particular interest in *C9orf72* mutation carriers,^13-15^ to be further subdivided. Furthermore, the tool outputs DTI metrics for the segmented thalamic subregions, such as mean diffusivity (MD; the overall magnitude of diffusion in a voxel), which can be used to additionally probe changes in their underlying tissue properties.

In this study, our aim was to investigate both thalamic subregional volumetric and microstructural changes using this novel thalamus parcellation tool on cross-sectional data from the large GENFI cohort to identify which subregions are commonly and differentially affected at presymptomatic and symptomatic stages across the three main genetic groups in FTD.

## Materials and methods

### Participants

We analysed data from the second phase of the GENFI study (GENFI2) available at the time of the sixth data freeze (03 March 2015–31 January 2021). In total, 994 participants across 25 sites (in the UK, Canada, Italy, the Netherlands, Sweden, Portugal, Germany, France, Spain and Belgium) took part in this phase. All aspects of the study were approved by the local ethics committee for each GENFI site. Written informed consent was obtained from all participants.

**Figure 1:**
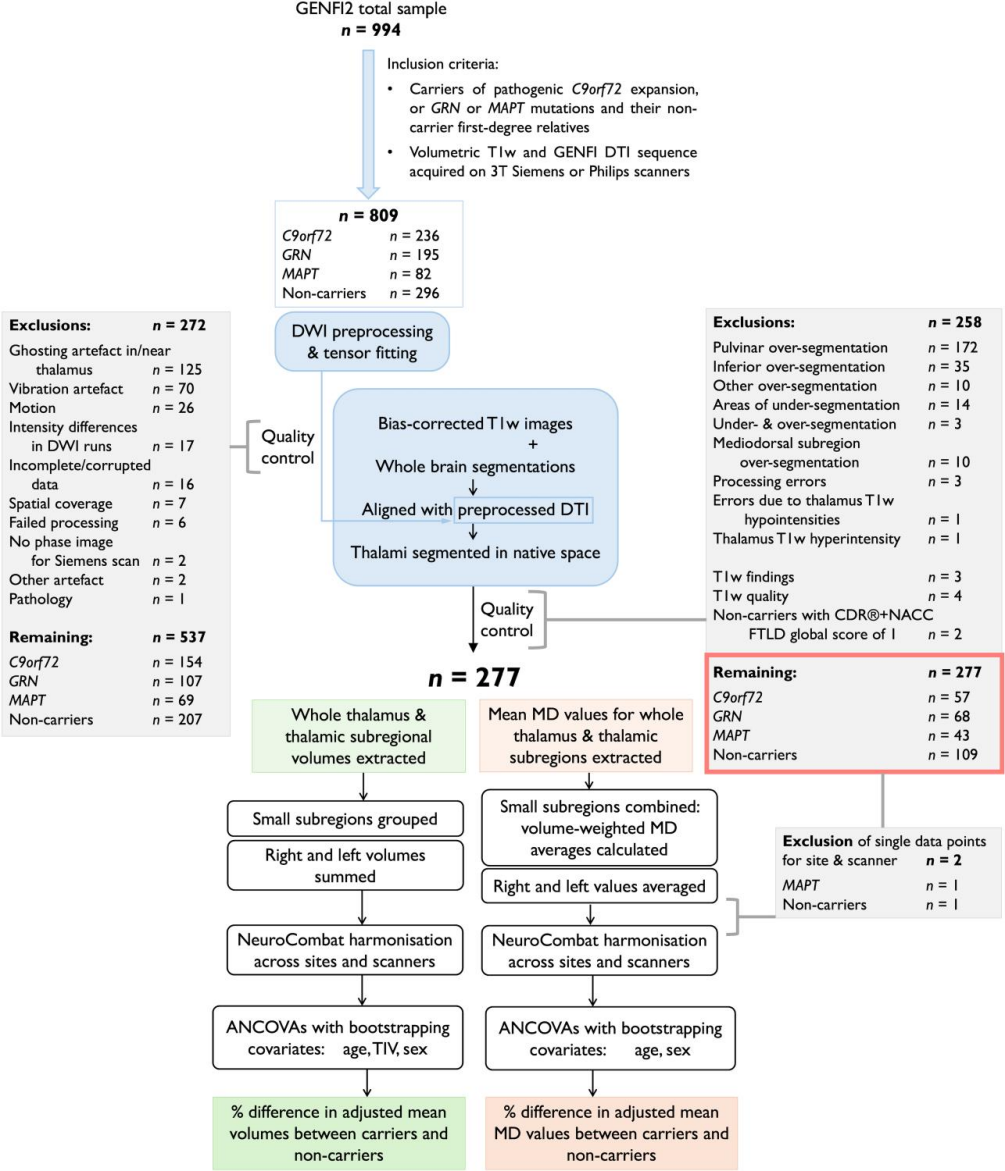
**Overview of participant selection, data quality control and processing steps.** ANCOVA, analysis of covariance; DWI, diffusion-weighted MRI; DTI, diffusion tensor imaging; GENFI, GENetic Frontotemporal dementia Initiative; GENFI2, the second phase of the GENFI study; MD, mean diffusivity; T1w, T1-weighted MRI; TIV, total intracranial volume.

Participants were screened and genotyped at their local sites for the pathogenic genetic mutations for FTD. We included participants who were carriers of pathogenic *C9orf72* hexanucleotide repeat expansion, or *GRN* or *MAPT* mutations, and their non-carrier first-degree relatives who acted as controls within the study. Participants from families with mutations in rarer FTD disease-causing genes were not included due to small numbers. We selected mutation carriers and non-carriers who had a volumetric T1w and a diffusion-weighted (DWI) MRI scan acquired with the standardized GENFI2 DTI sequence on 3T Siemens (Prisma, Skyra or Trio) or Philips Achieva scanners (*n* = 809). We carried out comprehensive imaging data quality control (QC) and analysed data from the participants’ first visits for which the data passed the quality checks (*n* = 277). Although our QC procedure led to exclusion of a large number of participants, we considered it necessary to ensure accuracy of the results. Participant selection, data QC and processing steps are summarized in Fig. 1 and further details are given in the following sections.

### MRI acquisition

Participants underwent a volumetric T1w MRI scan at an isotropic resolution of 1.1 mm. DWI data were acquired with a resolution of 2.5 × 2.5 × 2.5 mm^3^ and two identical repetitions, each with 64 diffusion directions at a *b*-value of 1000 s/mm^2^. Details of MRI acquisition parameters are given in Bocchetta *et al*.^9^

### Processing of imaging data

#### T1w data processing

T1w images were first bias-field corrected with the N4 method^16^ via geodesic information flow (GIF) processing^17^ and then parcellated using SynthSeg-robust^18,19^ (https://surfer.nmr.mgh.harvard.edu/fswiki/SynthSeg). Total intracranial volume (TIV) was obtained with SPM12 (Statistical Parametric Mapping, Wellcome Trust Centre for Neuroimaging, London, UK) running on Matlab2014b based on participants’ T1w scans from their initial GENFI2 visit.

#### DWI processing

We preprocessed the DWI data as described previously^9^ using a pipeline implemented in Nipype (https://nipype.readthedocs.io/en/latest/). In short, the DWI acquisitions were combined, aligned to an averaged *b* = 0 image and corrected for motion and eddy current distortions^20^ using the FMRIB Software Library (FSL) v5.0.10 tools.^21^ Susceptibility-induced distortions were corrected either via the unified phase unwrapping and T1w image registration scheme^22^ for Siemens data, or via non-linear registrations with T1w images for Philips data. Tensors were fitted on the eddy-, motion- and susceptibility-corrected data using NiftyFit.^23^ Data from one or both DWI repetitions were used for DTI processing, depending on their availability and quality.

Data were visually inspected for quality and artefacts, leading to initial exclusion of 272 participants due to issues such as motion, vibration, ghosting or other artefacts, incomplete spatial coverage and incomplete data (Fig. 1).

#### Thalamus segmentations

We segmented thalamic subregions using the segmentation tool that jointly incorporates structural and diffusion MRI data^12^ (https://surfer.nmr.mgh.harvard.edu/fswiki/ThalamicNucleiDTI).

Diffusion tensors were first ‘cleaned’ by replacing infeasible tensors (i.e. those with one or more negative eigenvalues) with a local mean value from Gaussian kernel convolution in the logarithmic domain.^24^ The eigenvectors, eigenvalues and FA were then derived from the ‘cleaned’ tensors.^12^ Bias-corrected T1w images and whole-brain parcellations were aligned to the diffusion data using an affine transform generated during DWI preprocessing, and the FA map that had been upsampled to the resolution of 1 × 1 × 1 mm^3^ as the reference image. We use the aligned structural images, together with the FA map (at original resolution), eigenvectors and eigenvalues as the input to the thalamus segmentation tool.

All thalamus segmentations were visually inspected, and 245 participants were excluded due to segmentation inaccuracies (Fig. 1). The most common reasons for exclusion were pulvinar overextension in the medial direction and/or into the fornix^12,25^ and over-segmentation of the thalamus in the inferior direction. We excluded data from a further three participants due to processing errors, four participants due to poor T1w data quality, three participants with pathological findings on T1w images unlikely to be related to FTD, one participant with marked thalamus T1w hyperintensity and two non-carriers with CDR® plus NACC FTLD global score of 1.

**Figure 2:**
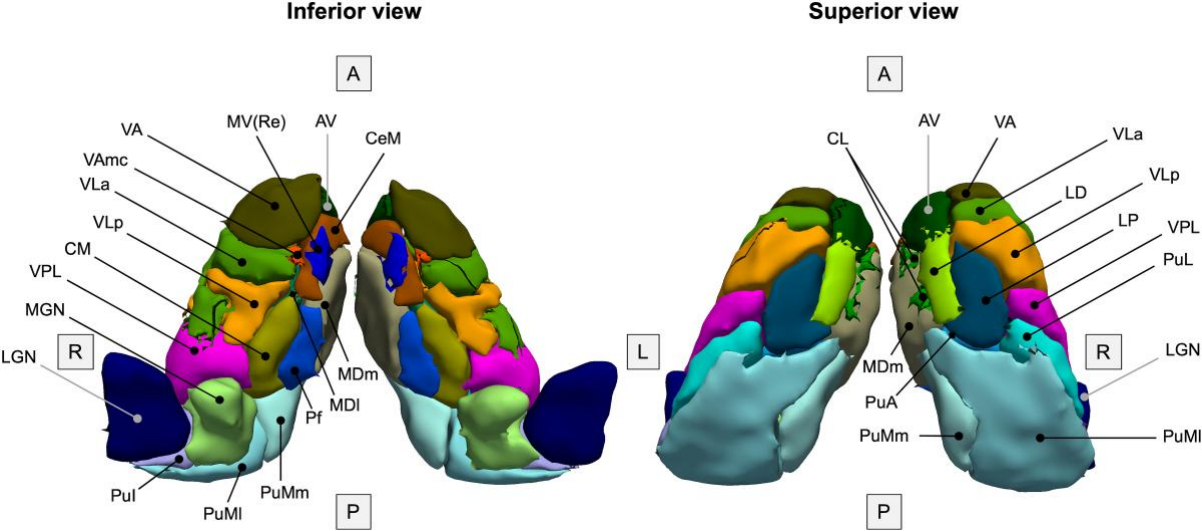
**Thalamus segmentation atlas.** Inferior and superior views of bilateral thalami are shown with the subregions of the right thalamus labelled. Note that a discretized version of the atlas is shown for visualization purposes; the thalamus segmentation tool uses a probabilistic atlas. The discretized atlas was created by taking the label with the highest probability, as per the probabilistic atlas, at every spatial location. Reticular and limitans subregions, which are not included in the current analysis, are not displayed. Also not visible are the ventromedial (VM), paracentral (Pc) and paratenial (Pt) subregions due to the discretized nature of the displayed atlas. Corresponding full names for the abbreviations can be found in Table 1. A, anterior; L, left; P, posterior; R, right.

Volumes of 27 right and left thalamic subregions were extracted from segmentation posterior probabilities by the segmentation tool. Figure 2 shows the discretized thalamus segmentation atlas to illustrate spatial locations of the thalamic subregions. We excluded the reticular and limitans subregions, grouped small subregions together (Table 1), and summed the right and left volumes. We followed the grouping by Bocchetta *et al*.^26^ except for the pulvinar, where we analysed its two medial subdivisions separately. The medial pulvinar was included within the total pulvinar in the original grouping by Bocchetta *et al*.^26^ but could be further subdivided here due to additional information from the diffusion data. Volumes of the resulting 16 thalamic regions and the whole thalamus were then harmonized across sites and scanners using NeuroCombat version 0.2.12 in Python^27,28^ while preserving variability due to the genetic group with disease stage, age at visit, sex and TIV. We divided sites into distinct ‘site-scanner’ combinations so that sites with multiple scanner types (Siemens Prisma, Skyra or Trio, or Philips Achieva) were split according to the scanner used and treated separately in the data harmonization.

**Table 1:** Grouping of thalamic subregions for volumetric and mean diffusivity analyses

Segmentation subregions	(abbreviation)	Grouping for volumes	Grouping for mean diffusivity
Anteroventral	(AV)	AV	AV
Laterodorsal	(LD)	LD	LD
Lateral posterior	(LP)	LP	LP
Ventral anterior	(VA)	VA	VAM
Ventral anterior magnocellular	(VAmc)		
Ventromedial	(VM)	VM	
Ventral lateral anterior	(VLa)	VLa	VLa
Ventral lateral posterior	(VLp)	VLp	VLp
Ventral posterolateral	(VPL)	VPL	VPL
Central medial	(CeM)	Intralaminar	Intralaminar
Central lateral	(CL)		
Paracentral	(Pc)		
Centromedian	(CM)		
Parafascicular	(Pf)		
Paratenial	(Pt)	Midline	Medial
Reuniens	(MV-re)		
Mediodorsal medial magnocellular	(MDm)	MeD	
Mediodorsal lateral parvocellular	(MDl)		
Lateral geniculate	(LGN)	LGN	LGN
Medial geniculate	(MGN)	MGN	MGN
Pulvinar medial, medial part	(PuMm)	PuMm	PuMm
Pulvinar medial, lateral part	(PuMl)	PuMl	PuMl
Pulvinar anterior	(PuA)	Non-medial pulvinar	Non-medial pulvinar
Pulvinar lateral	(PuL)		
Pulvinar inferior	(PuI)		

Abbreviations: VAM, ventral anterior and ventromedial region; MeD, mediodorsal region.

Mean MD values of the segmented subregions were derived by the segmentation tool from diffusion tensors interpolated in the logarithm domain^24^ and were weighted by segmentation posterior probabilities.^12^ These two steps help to mitigate the impact of the relatively low resolution of the DTI data on the derived measures. We grouped small subregions together (Table 1), calculated volume-weighted average MD values for the grouped ipsilateral regions and averaged these across the right and left sides. Grouping was performed more coarsely than for volumes to increase robustness for the noisier MD measure. Specifically, we grouped the ventromedial with the ventral anterior subregions, and the mediodorsal (MeD) subregions with the paratenial and reuniens (i.e. midline) subregions, due to their respective anatomical groupings and spatial proximity.^11^ The resulting MD values for 14 thalamic regions and the whole thalamus were harmonized across sites and scanners separately to volumes using NeuroCombat while preserving the variability due to the genetic group with disease stage, age at visit and sex.

Prior to harmonization of volumes and MD values, we excluded two further participants who were the only individuals at their respective sites to have had imaging data acquired on a particular scanner after the data and segmentation QC. The final study cohort consisted of 277 participants (57 *C9orf72* expansion carriers, 68 *GRN* mutation carriers, 43 *MAPT* mutation carriers and 109 mutation non-carriers) from 18 sites (Table 2).

**Table 2:** Demographic and clinical summary for the study participants

Genetic group	Non-carriers	*C9orf72* expansion carriers		*GRN* mutation carriers		*MAPT* mutation carriers	
CDR®+NACC FTLD global score		≤0.5	≥1	≤0.5	≥1	≤0.5	≥1
*N*	109	47	10	57	11	31	12
Age, years [median (IQR)]	41.4 (17.8)	37.1 (13.1)*	60.6 (10.6)**	43.5 (18.2)	61.7 (9.2)**	36.0 (13.6)	59.2 (20.8)*
Sex, *n* male (%)	42 (39%)	17 (36%)	8 (80%)*	22 (39%)	4 (36%)	13 (42%)	5 (42%)
Scanners, *n* (%)							
Siemens Trio	22 (20%)	11 (23%)	0 (0%)	16 (28%)	3 (27%)	7 (23%)	3 (25%)
Siemens Skyra	20 (18%)	7 (15%)	2 (20%)	15 (26%)	4 (36%)	3 (10%)	1 (8%)
Siemens Prisma	53 (49%)	25 (53%)	7 (70%)	22 (39%)	4 (36%)	15 (48%	8 (67%)
Philips Achieva	14 (13%)	4 (9%)	1 (10%)	4 (7%)	0 (0%)	6 (19%)	0 (0%)
Clinical phenotype, *n*	n/a	n/a	7 bvFTD, 2 FTD-ALS, 1 Other	n/a	5 bvFTD, 5 PPA, 1 CBS	n/a	9 bvFTD, 1 PPA, 1 Dementia-NOS, 1 Other

Abbreviations: bvFTD, behavioural variant frontotemporal dementia; CBS, corticobasal syndrome; FTD-ALS, frontotemporal dementia-amyotrophic lateral sclerosis; IQR, interquartile range; n/a, not applicable; NOS, not otherwise specified; PPA, primary progressive aphasia. ‘Other’ indicates no clear diagnosis. *P < 0.05, **P < 0.001 compared with non-carriers on Mann–Whitney U-test (for differences in age) or Fisher’s exact test (for differences in sex).

### Clinical assessment

All participants completed a standardized clinical assessment as detailed previously.^4^ We divided carriers in each genetic group further according to their CDR® plus NACC FTLD global score.^29^ Carriers with a global score of 0 or 0.5 were considered to be at the presymptomatic or prodromal stage and were grouped together into a single ‘presymptomatic’ group, while carriers with a global score of 1 and above were considered to be symptomatic. Demographic and clinical information is shown in Table 2.

Symptomatic mutation carriers in all three genetic groups were significantly older (*P* ≤ 0.01, Mann–Whitney U-tests), while presymptomatic *C9orf72* expansion carriers were younger (*P* = 0.015, Mann–Whitney U-test) compared with mutation non-carriers. There were more males in the symptomatic *C9orf72* group compared with the non-carrier group (*P* = 0.017, Fisher’s exact test). Scanner type did not differ significantly between mutation non-carriers and each presymptomatic (*P* ≥ 0.246, chi-square tests) or symptomatic (*P* ≥ 0.335, Fisher–Freeman–Halton exact tests) genetic group.

### Statistical analysis

Harmonized thalamic regional volumes and MD values for presymptomatic and symptomatic carriers in each genetic group were compared to those of mutation non-carriers using analyses of covariance (ANCOVA) with bias-corrected and accelerated (BCa) bootstrapping (2000 samples) in SPSS version 28.0 software (SPSS Inc., Chicago, IL, USA). We included age, sex and TIV as covariates for the volume comparisons, and age and sex as covariates for the MD comparisons. Correction for multiple comparisons was carried out using the Benjamini–Hochberg method^30^ with a 5% false discovery rate (FDR) in each genetic group separately for volume and MD comparisons. We considered *P*-values from ANCOVA pairwise comparisons between mutation carriers and non-carriers, as well as between presymptomatic and symptomatic carriers when performing the FDR correction.

We calculated percentage differences in the adjusted volumes and MD values relative to non-carriers for each carrier group using the estimated marginal means (EMM) from the respective ANCOVAs:


$$\mathrm{percentage}\;\mathrm{difference}\;(\text{\%})=\frac{\mathrm{EMM}\;\mathrm{non}\text{-}\mathrm{carriers}\;\text{–}\;\mathrm{EMM}\;\mathrm{carriers}}{\mathrm{EMM}\;\mathrm{non}\text{-}\mathrm{carriers}}\;\times \;100$$

A *positive* percentage difference thus indicates a *lower* adjusted mean for mutation carriers relative to non-carriers, while a *negative* value indicates a *higher* adjusted mean for mutation carriers compared with non-carriers.

## Results

**Figure 3:**
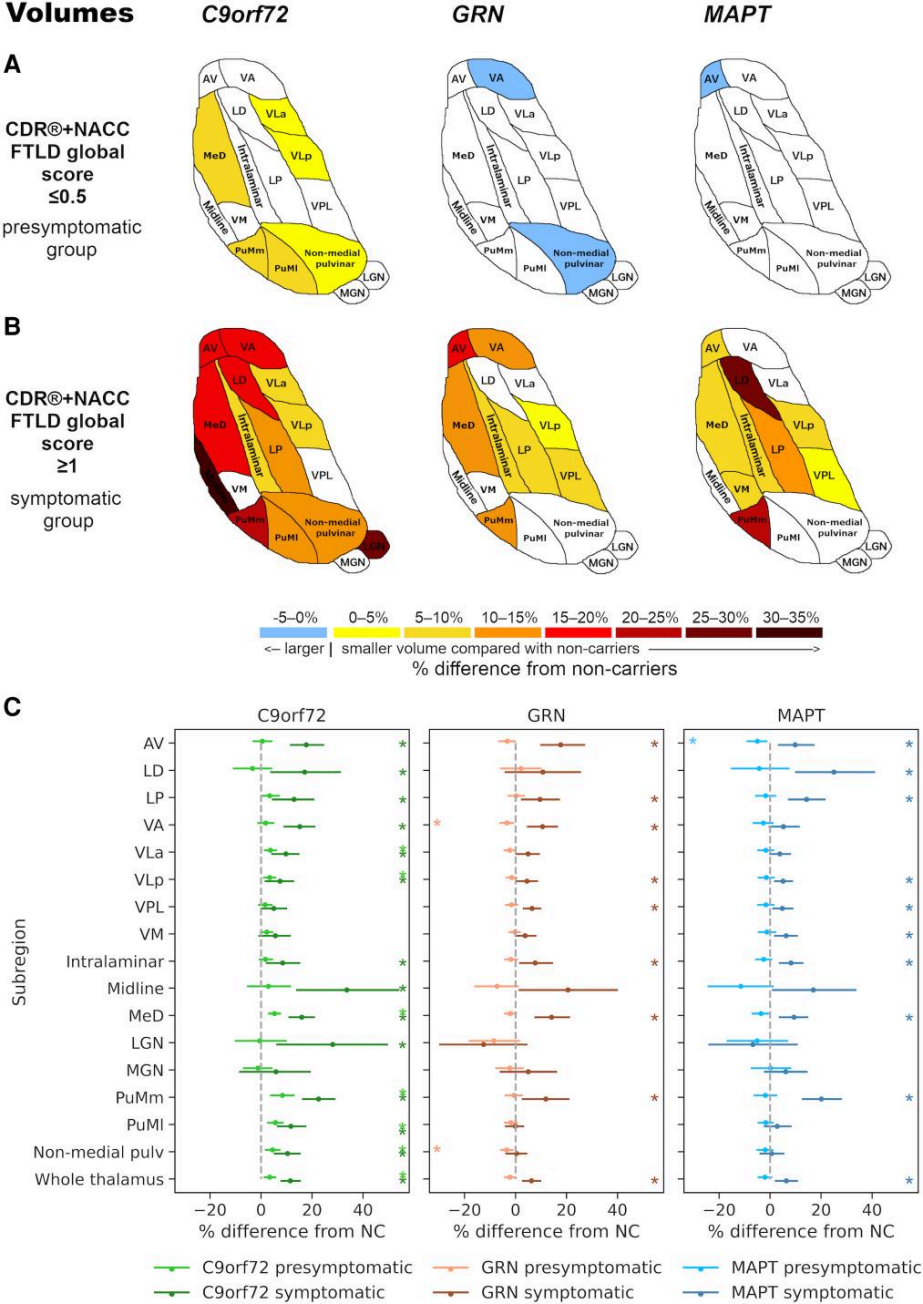
**Thalamic subregional volumetric differences in presymptomatic (panel A) and symptomatic (panel B) mutation carriers for *C9orf72*, *GRN* and *MAPT* groups compared with non-carriers.** The colour bar indicates the percentage differences: positive values indicate smaller (right and left summed) volumes while negative values indicate larger volumes in presymptomatic (47 *C9orf72*, 57 *GRN*, 31 *MAPT*) and symptomatic (10 *C9orf72*, 11 *GRN*, 12 *MAPT*) mutation carriers relative to 109 non-carriers on univariate analyses of covariance (ANCOVAs) for each subregion and genetic group, followed by pairwise comparisons. Volume differences for all coloured subregions in panels **A** and **B** are significant at *P* < 0.05 after correction for multiple comparisons using the Benjamini–Hochberg method. (**C**) Plots showing point estimates (circles) and 95% CIs (lines) for the percentage differences in volumes between mutation carriers and non-carriers. The shown 95% CIs are the bootstrapped upper and lower 95% CI limits for the raw group differences in EMMs (non-carriers − carriers; Supplementary Table 1) expressed as a percentage of the corresponding EMM for non-carriers (e.g. (95% CI lower limit for [non-carriers’ EMM − carriers’ EMM]/non-carriers’ EMM) × 100), to complement calculation of the point estimates. Asterisks denote significance at *P* < 0.05 after correction for multiple comparisons. NC, non-carrier. Thalamic subregions: AV, anteroventral; LD, laterodorsal; LGN, lateral geniculate; LP, lateral posterior; MeD, mediodorsal; MGN, medial geniculate; pulv, pulvinar; PuMl, lateral part of the medial pulvinar; PuMm, medial part of the medial pulvinar; VA, ventral anterior; VLa, ventral lateral anterior; VLp, ventral lateral posterior; VM, ventromedial; VPL, ventral posterolateral.

**Figure 4:**
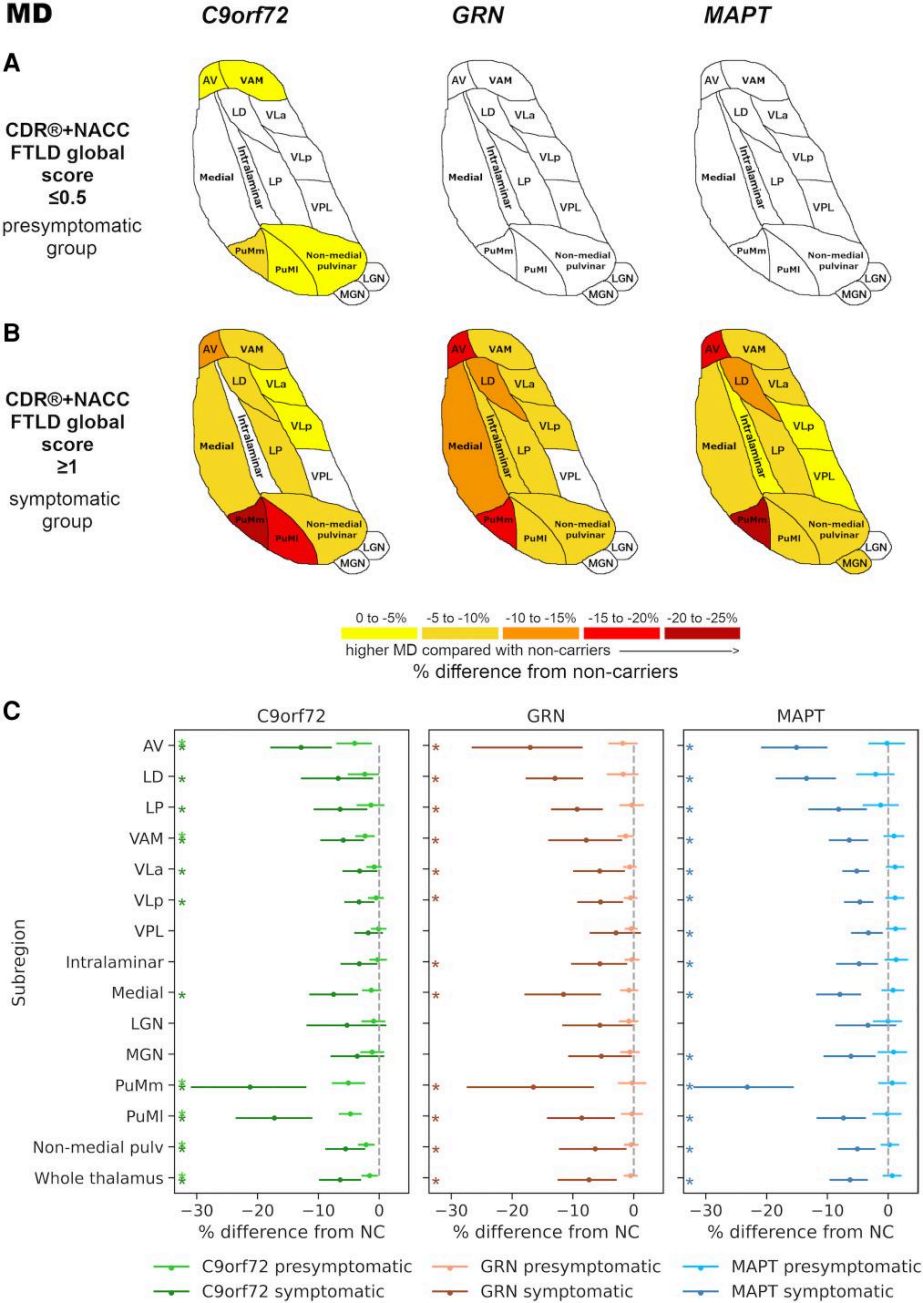
**Higher MD of thalamic subregions in presymptomatic (panel A) and symptomatic (panel B) mutation carriers for *C9orf72*, *GRN* and *MAPT* groups compared with non-carriers.** The colour bar indicates the percentage differences in MD: negative values indicate higher (right and left averaged) MD values in presymptomatic (47 *C9orf72*, 57 *GRN*, 31 *MAPT*) and symptomatic (10 *C9orf72*, 11 *GRN*, 12 *MAPT*) mutation carriers relative to 109 non-carriers on univariate analyses of covariance (ANCOVAs) for each subregion and genetic group, followed by pairwise comparisons. The MD differences for all coloured regions in panels **A** and **B** are significant at *P* < 0.05 after correction for multiple comparisons using the Benjamini–Hochberg method. (**C**) Plots showing point estimates (circles) and 95% CIs (lines) for the percentage differences in MD between mutation carriers and non-carriers. The shown 95% CIs are the bootstrapped upper and lower 95% CI limits for the raw group differences in EMMs (non-carriers – carriers; Supplementary Table 2) expressed as a percentage of the corresponding EMM for non-carriers (e.g. (95% CI lower limit for [non-carriers’ EMM – carriers’ EMM]/non-carriers’ EMM) × 100), to complement calculation of the point estimates. Asterisks denote significance at *P* < 0.05 after correction for multiple comparisons. NC, non-carrier. Thalamic subregions: AV, anteroventral; LD, laterodorsal; LGN, lateral geniculate; LP, lateral posterior; MGN, medial geniculate; pulv, pulvinar; PuMl, lateral part of the medial pulvinar; PuMm, medial part of the medial pulvinar; VAM, ventral anterior and ventromedial; VLa, ventral lateral anterior; VLp, ventral lateral posterior; VPL, ventral posterolateral.

Overall, all three genetic groups showed significant differences in thalamic subregional volumes and MD values compared with non-carriers at the symptomatic stage, whilst presymptomatic involvement was evident mainly in *C9orf72* expansion carriers (Figs 3 and 4). The EMMs, differences in EMMs, corresponding 95% confidence intervals (CI) and *P*-values from ANCOVAs for all measures are reported in Supplementary Tables 1 and 2. Specific results for volumes and MD values are reported below.

### Volumes

#### *C9orf72* expansion carriers

Significantly smaller volumes were found in the presymptomatic *C9orf72* group compared to non-carriers in several thalamic subregions, which were most pronounced in the PuMm, PuMl and MeD (5–8% volumetric difference from non-carriers, corrected *P* < 0.01) with 3–4% difference in the remaining affected subregions (non-medial pulvinar, VLa and VLp; corrected *P* < 0.01) (Fig. 3A and C). At the symptomatic stage, the volume differences were more widespread and severe, where all regions except for the VM, VPL and MGN showed at least 7% smaller volumes than non-carriers (corrected *P* ≤ 0.035). The differences were most prominent in the midline subregion (34%, corrected *P* < 0.01), the LGN (28%, corrected *P* = 0.014) and the PuMm (23%, corrected *P* < 0.01) (Fig. 3B and C).

The volume of the whole thalamus was significantly smaller in carriers at both the presymptomatic (3%) and the symptomatic (12%) stage compared with non-carriers (corrected *P* < 0.01) (Fig. 3C).

#### *GRN* mutation carriers

In presymptomatic *GRN* mutation carriers, no thalamic subregions had significantly smaller volumes than non-carriers, and volumes of the non-medial pulvinar (−3%, corrected *P* < 0.01) and the VA subregion (−3%, corrected *P* = 0.042) were larger compared with non-carriers (Fig. 3A and C). In contrast, volumes of several subregions were significantly smaller compared with non-carriers at the symptomatic stage, most notably in the AV and MeD (18% and 14%, respectively, corrected *P* < 0.01), and the PuMm (12%, corrected *P* = 0.028) (Fig. 3B and C), followed by 4–11% difference for the VA, LP, intralaminar, VPL and VLp subregions (corrected *P* ≤ 0.036).

The whole thalamus showed statistically significant smaller volume than non-carriers at the symptomatic stage (6%, corrected *P* < 0.01) (Fig. 3C).

#### *MAPT* mutation carriers

The only significant difference from non-carriers in the presymptomatic *MAPT* group was a larger AV volume (−5%, corrected *P* = 0.043) (Fig. 3A and C). Several subregions showed smaller volumes at the symptomatic stage compared with non-carriers, which were most pronounced for the LD (25%), PuMm (20%) and LP (14%) (corrected *P* < 0.01), followed by 5–10% difference for the AV, MeD, intralaminar, VM, VLp and VPL subregions (corrected *P* ≤ 0.032) (Fig. 3B and C).

Whole thalamus volume was significantly smaller in the symptomatic *MAPT* group compared with non-carriers (6%, corrected *P* < 0.01) (Fig. 3C).

### Mean diffusivity

#### *C9orf72* expansion carriers

Presymptomatic *C9orf72* expansion carriers showed significantly higher MD values than non-carriers, which were most pronounced for the PuMm and PuMl (−5%, corrected *P*  *<* 0.01), and the AV (−4%, corrected *P* = 0.011), followed by a small (−2%) difference for the VAM and non-medial pulvinar (corrected *P*  *<* 0.01) (Fig. 4A and C). The PuMm, PuMl and AV also showed the largest differences in MD values at the symptomatic stage compared with non-carriers (−21%, −17% and −13%, respectively; corrected *P* < 0.01), followed by significantly higher MD values in most other subregions (−7 to −3% for the medial subregion, LD, LP, VAM, non-medial pulvinar, VLa and VLp; corrected *P* ≤ 0.030) (Fig. 4B and C).

The MD of the whole thalamus was significantly higher in both presymptomatic (−2%, corrected *P* = 0.024) and symptomatic (−6%, corrected *P* < 0.01) carriers compared with non-carriers (Fig. 4C).

#### *GRN* mutation carriers

No significant differences in MD were found for presymptomatic *GRN* mutation carriers compared with non-carriers. At the symptomatic stage, MD values were significantly higher than non-carriers in all subregions except for the VPL, MGN and LGN, with at least −5% difference (corrected *P* ≤ 0.046). The differences were most prominent in the AV (−17%), PuMm (−16%), and LD (−13%) (corrected *P* < 0.01) (Fig. 4B and C).

Higher MD of the whole thalamus compared to non-carriers was statistically significant at the symptomatic stage (−7%, corrected *P* < 0.01) (Fig. 4C).

#### *MAPT* mutation carriers

*MAPT* mutation carriers showed higher MD values than non-carriers only at the symptomatic stage, where the MD was significantly higher in all subregions except for the LGN, and the difference most pronounced in the PuMm (−23%), AV (−15%) and LD (−13%) (corrected *P* < 0.01). The difference in MD ranged from −8 to −3% for the rest of subregions (corrected *P* < 0.01) (Fig. 4B and C).

The MD of the whole thalamus was significantly higher at the symptomatic stage in the *MAPT* group compared with non-carriers (−6%, corrected *P* < 0.01) (Fig. 4C).

### Subgroup analysis

Due to group differences in age, we compared subregional and whole thalamic volumes together with MD values between symptomatic mutation carriers and mutation non-carriers over the age of 52 years. The subanalysis included the same 10 *C9orf72* expansion carriers (age range: 53–74 years) and 11 *GRN* mutation carriers (age range: 53–76 years) at the symptomatic stage as in the main analysis, and a subgroup of nine symptomatic *MAPT* mutation carriers (age range: 54–68 years; three individuals excluded due to age < 52 years) and 28 mutation non-carriers (age range: 53–79 years).

A very similar pattern of the most prominent differences in volumes (Supplementary Fig. 1A) and MD (Supplementary Fig. 1B) remained for symptomatic *C9orf72* and *MAPT* mutation carriers compared with mutation non-carriers, as found in the main analysis. The results for the symptomatic *GRN* group also remained similar, however, now bordered significance for the differences in PuMm volume (corrected *P* = 0.051) and MD (corrected *P* = 0.055) compared with non-carriers.

## Discussion

This is the first study to utilize T1w imaging and DTI jointly to characterize thalamic subregional involvement in the three main genetic groups in FTD. Following detailed data QC, we detected presymptomatic involvement of the thalamus in *C9orf72* expansion carriers relating to both volumetric and diffusion measures, and subtly larger thalamic subregional volumes in presymptomatic *GRN* and *MAPT* mutation carriers compared with non-carriers. At the symptomatic stage, the most affected subregions in each genetic group were those with associative and limbic functions, which is compatible with FTD symptomatology. We assessed subdivisions of the medial pulvinar for the first time and identified prominent involvement of the most medial part of the medial pulvinar (PuMm), as defined by the segmentation tool, to be a common feature in symptomatic mutation carriers across the genetic groups.

We found that smaller thalamic volumes were most marked and widespread in *C9orf72* expansion carriers, in whom they were already present at the presymptomatic stage. Our findings are in line with those from thalamic segmentations using T1w imaging only,^7,9,26^ where presymptomatic atrophy of the pulvinar as a whole was identified as a prominent feature in *C9orf72* expansion carriers.^9^ Here, we confirmed presymptomatic involvement of pulvinar subdivisions in the *C9orf72* group, finding both volumetric and MD differences compared with non-carriers. The largest volume difference for presymptomatic *C9orf72* expansion carriers was in the PuMm, which was also one of the most affected regions at the symptomatic stage in this genetic group. This is consistent with medial pulvinar atrophy in both presymptomatic and symptomatic *C9orf72* expansion carriers reported by Lee *et al*.^14,15^ using voxel-based morphometry. It is also supported by *post mortem* observations of the medial pulvinar being a site of notable gliosis and multiple types of *C9orf72*-associated neuronal inclusions,^13^ and of its (albeit not statistically significant) volume reductions^31^ in *C9orf72* expansion carriers. Our finding of higher MD values in the PuMm suggests that volume reductions are accompanied by underlying changes in tissue microstructure.

The PuMm was one of the most affected regions also in symptomatic *GRN* and *MAPT* mutation carriers in both volumetric and MD analyses. The pulvinar is a large, associative nuclear complex, which can be subdivided further into anterior, lateral, inferior and medial parts, each with different roles and connections.^32,33^ The medial pulvinar has connections with the prefrontal, cingulate, superior temporal and sensory association cortices, its most medial part also being connected with the amygdala.^32-35^ As such, the function of the medial pulvinar is thought to be in attentional processing and integration of multisensory with limbic information.^32,36^ It has also been proposed that the medial pulvinar is involved in recognition of fearful facial expressions.^32,37^ However, the role of the medial pulvinar remains understudied^38^ and is yet to be fully established. Although symptomatic mutation carriers in the GENFI cohort show difficulties with facial emotion recognition, which are already detectable at the late presymptomatic stage in *C9orf72* expansion carriers for negative emotions, correlations between these deficits and thalamic grey matter density are not consistent across the genetic groups.^39^ Further work is therefore needed to assess cognitive correlates of medial pulvinar atrophy in genetic FTD.

In contrast to the *C9orf72* group, presymptomatic *GRN* mutation carriers showed subtly (−3%) larger VA and non-medial (combined anterior, lateral and inferior) pulvinar volumes relative to non-carriers. Similarly, *MAPT* carriers had a larger AV volume (−5%) compared with non-carriers. These findings are unexpected as significantly larger volumes of thalamic subregions have not been found by previous thalamus segmentation studies. The reason for the larger subregional volumes in presymptomatic *GRN* and *MAPT* mutation carriers is unclear and cannot be attributed with certainty to either pathophysiological effects or methodological issues. Given the small magnitude of the volume differences and our sample size, a replication in a larger sample is needed to confirm if these are true effects.

At the symptomatic stage, the three genetic groups had pronounced atrophy of the PuMm in common, but otherwise differed in the most atrophied subregions. These were the midline and the LGN in the *C9orf72* group (albeit with large confidence intervals), the AV and the MeD in the *GRN* group and the LD and LP in the *MAPT* group. Except for the LGN, all these subregions have associative and/or limbic functions^26^ and their volume reductions in genetic FTD have been reported previously.^40^

While several segmentation studies have found LGN volume reductions in symptomatic *C9orf72* expansion carriers,^7,9,26^ one study did not corroborate this finding in *C9orf72*-associated FTD-ALS.^8^ The LGN relays visual information, and it has been proposed that its atrophy may be related to visual hallucinations that *C9orf72* expansion carriers experience.^26^ The MeD, one of the most affected subregions in symptomatic *GRN* mutation carriers, has reciprocal connections with the dorsolateral prefrontal cortex,^33^ which is affected early^7^ and severely^9^ in this genetic group. Due to its cortical connections, the MeD is a part of complex circuits involved in executive, cognitive and emotional processes.^33^ Similarly, the AV is a limbic subregion connected with the hippocampal formation and the amygdala,^41^ structures which also exhibit prominent atrophy in symptomatic *GRN* mutation carriers.^9^ Similarly to the AV, the LD is a limbic subregion with connections with the hippocampal formation,^33^ which is amongst the earliest^4,7,9^ and most severely^9^ affected regions in *MAPT* mutation carriers. On the other hand, the LP has connections with the parietal cortex^5^ and plays a role in higher-order somatosensory and visuospatial integration.^41^ It is less clear how its prominent volumetric involvement relates to typical symptoms or involvement of other brain regions in symptomatic *MAPT* mutation carriers.

Previous segmentation studies using T1w imaging only have reported more widespread atrophy of thalamic subregions in symptomatic mutation carriers^7,9^ compared with our findings. These differences could be due to our smaller sample size, the stringent quality control protocol we adopted in this study, or the differences in the thalamus segmentation method used.

On MD analyses, the most marked involvement of the PuMm and AV was a shared feature across all three genetic groups at the symptomatic stage. The MD increases were more widespread than the detected atrophy in the symptomatic *GRN* and *MAPT* groups, but not for *C9orf72* expansion carriers. In the context of neurodegenerative diseases, increases in grey matter MD at symptomatic stages are thought to reflect loss of neurons and dendrites, allowing water molecules to diffuse more freely.^42^ When detected at presymptomatic stages, grey matter MD increases have been suggested to indicate early neuronal loss,^43^ while MD decreases could signify the preceding inflammation and cellular swelling restricting water diffusion.^44,45^ Unlike volumetric measurements, however, we did not detect significant presymptomatic MD differences in *GRN* and *MAPT* groups compared with non-carriers. The MD therefore did not appear to be overall more sensitive than volumes in detecting thalamic subregional involvement, which could also be due to diffusion indexes not being able to pick up very early tissue changes. This is in contrast to studies showing that DTI changes are detectable earlier than atrophy in FTD.^9,46,47^ While early white matter diffusion changes may be evident on DTI measures, more advanced diffusion imaging models may be better suited for the assessment of underlying tissue property changes in subcortical grey matter.^48^

The main limitation of this study is the small sample size for mutation carriers. While we consider rigorous QC necessary to ensure accurate results and as one of the strengths of our analyses, it did lead to exclusion of a large number of participants. This was particularly the case for *C9orf72* expansion carriers, in whom over-segmentation of the pulvinar was a common issue. Our results may consequently be underestimating, or alternatively overestimating, the degree and extent of thalamic involvement, as the likelihood of passing segmentation QC may be associated with the degree of atrophy. MRI data quality and the tissue contrast within the images are other potential factors affecting thalamic segmentation accuracy, which could in turn be related to the clinical status if these are degraded by subtle motion artefacts. Perhaps future studies with a more lenient threshold for quality acceptance of the segmentations could establish whether such strict QC is necessary to reliably detect differences in thalamus subregions between clinical groups. In addition, the thalamus segmentation tool has been developed further using a convolutional neural network,^25^ which may help alleviate the segmentation inaccuracies encountered in our analyses and thus increase sample sizes in future studies. Moreover, although the automated tool was previously validated by Tregidgo *et al.*^12^ in independent cohorts of healthy individuals and patients with Alzheimer's disease, ensuring its general reliability, a definitive exclusion of any potential impact of FTD-specific pathology on segmentation performance would ideally require validation within each genetic FTD group. This would involve comparing automated outputs against manual segmentations across all thalamic subregions. However, given the substantial time and resource demands of such procedures and our detailed visual quality inspection of all segmentations, this level of validation was beyond the scope of the present study.

Although we harmonized volume and MD measurements, we cannot exclude the possibility of residual scanner and site effects remaining due to small numbers and imbalances in disease stage and genetic groups for site-scanner combinations. Similarly, we controlled for age and sex (and TIV for volumetric comparisons) in our statistical analyses, but residual confounding effects may remain. Our findings therefore need replication in a larger cohort where harmonization and statistical analyses would be more robust.

When considering the size of the smaller thalamic subregions, the resolution of the source DWI is relatively low. The effect of this on subregional MD measurements is partly mitigated by the segmentation tool via deriving the MD from tensors interpolated to the voxel grid of the input T1w image.^12^ The MD measures however remain susceptible to partial volume effects that may artefactually raise MD values for subregions bordered by cerebrospinal fluid and have a disproportionate effect in the presence of atrophy.

The division between PuMm and PuMl in the thalamus segmentation tool atlas is based solely on the visible diffusion directionality contrast in the medial pulvinar on DTI,^12^ and not on borders derived from the histological atlas as is the case for other subregions. Therefore, the histological correspondence of these subdivisions remains to be confirmed, and the naming (PuMm and PuMl) used in this work refers to subdivisions defined by the segmentation tool rather than histology.

In this study, we only considered the combined left and right regions to limit the number of comparisons in a relatively small sample. We recognize that it is important to investigate the potential differences between left and right hemispheres in future studies, especially in *GRN* mutation carriers, given the characteristic pattern of brain asymmetry and its links with different disease course in this genetic group.^49^

Finally, we used cross-sectional data to detect thalamic subregional involvement at different disease stages and therefore cannot make inferences about which measures are most sensitive in detecting evolving pathological changes on an individual level. Future work could address this using longitudinal data from genetic FTD initiatives.

## Conclusion

We demonstrated thalamic subregional involvement in the three main genetic FTD groups by jointly utilising structural and diffusion MRI. Our findings using the novel method replicate results from previous studies, confirming that the involvement of thalamic subregions is a consistent pathological hallmark of genetic FTD. We additionally identified that the most medial part of the medial pulvinar is commonly affected across the genetic groups, warranting replication studies in larger cohorts and further research into associations between changes in the medial pulvinar and cognitive measures in genetic FTD.

## Supplementary Material

fcaf420_Supplementary_Data

## Data Availability

Anonymized data may be shared upon reasonable request from a qualified academic investigator for the purpose of replication of procedures and results detailed in this article.

## Appendix

### List of GENFI consortium authors

Rhian Convery, Sophie Goldsmith, Kiran Samra, Thomas Cope, Maura Malpetti, Antonella Alberici, Enrico Premi, Roberto Gasparotti, Emanuele Buratti, Valentina Cantoni, Andrea Arighi, Chiara Fenoglio, Vittoria Borracci, Maria Serpente, Tiziana Carandini, Emanuela Rotondo, Giacomina Rossi, Giorgio Giaccone, Giuseppe Di Fede, Paola Caroppo, Sara Prioni, Veronica Redaelli, David Tang-Wai, Ekaterina Rogaeva, Johanna Krüger, Miguel Castelo-Branco, Morris Freedman, Ron Keren, Sandra Black, Sara Mitchell, Christen Shoesmith, Robart Bartha, Rosa Rademakers, Jackie Poos, Janne M. Papma, Lucia Giannini, Liset de Boer, Julie de Houwer, Rick van Minkelen, Yolande Pijnenburg, Camilla Ferrari, Cristina Polito, Gemma Lombardi, Valentina Bessi, Enrico Fainardi, Stefano Chiti, Mattias Nilsson, Henrik Viklund, Melissa Taheri Rydell, Vesna Jelic, Abbe Ullgren, Tobias Langheinrich, Albert Lladó, Anna Antonell, Jaume Olives, Mircea Balasa, Nuria Bargalló, Sergi Borrego-Ecija, Ana Verdelho, Carolina Maruta, Tiago Costa-Coelho, Gabriel Miltenberger, Frederico Simões do Couto, Alazne Gabilondo, Ioana Croitoru, Mikel Tainta, Myriam Barandiaran, Patricia Alves, Benjamin Bender, David Mengel, Lisa Graf, Annick Vogels, Mathieu Vandenbulcke, Philip Van Damme, Rose Bruffaerts, Koen Poesen, Pedro Rosa-Neto, Maxime Montembault, Raffaella Migliaccio, Ninon Burgos, Daisy Rinaldi, Catharina Prix, Elisabeth Wlasich, Olivia Wagemann, Sonja Schönecker, Alexander Maximilian Bernhardt, Anna Stockbauer, Jolina Lombardi, Sarah Anderl-Straub, Adeline Rollin, Gregory Kuchcinski, Vincent Deramecourt, João Durães, Marisa Lima, Maria João Leitão, Maria Rosario Almeida, Miguel Tábuas-Pereira, Sónia Afonso and João Lemos.
