# Supplementary material for: Thalamus involvement in genetic frontotemporal dementia assessed using structural and diffusion MRI: a GENFI study
Source: Brain Commun. 2025 Oct 24;7(6):fcaf420. doi: 10.1093/braincomms/fcaf420 (PMC12612583; doi:10.1093/braincomms/fcaf420)
Supplement: fcaf420_Supplementary_Data [file fcaf420_supplementary_data.pdf]

Supplementary material

Supplementary Table 1. Thalamic subregional volume comparisons between mutation carriers and non-carriers. Positive differences indicate smaller (right and left summed) volumes while negative differences indicate larger volumes in mutation carriers relative to non-carriers. Uncorrected *p*-values are shown; asterisked bold *p*-values indicate significance at $p < 0.05$ after correction for multiple comparisons. The covariates included were age, sex and total intracranial volume. The 95% confidence interval limits for the percentage differences were calculated from the confidence interval limits for the raw group differences in estimated marginal means.

ANCOVA – univariate analysis of covariance, CI – confidence interval, EMM – estimated marginal mean from ANCOVA. Thalamic subregions: AV – anteroventral, LD – laterodorsal, LGN – lateral geniculate, LP – lateral posterior, MeD – mediodorsal, MGN – medial geniculate, PuMl – lateral part of the medial pulvinar, PuMm – medial part of the medial pulvinar, VA – ventral anterior, VLa – ventral lateral anterior, VLp – ventral lateral posterior, VM – ventromedial, VPL – ventral posterolateral.

Group			AV	LD	LP	VA	VLa	VLp	VPL	VM	Intralaminar	Midline	MeD	LGN	MGN	PuMm	PuMl	Non-medial pulvinar	Whole thalamus
C9orf72 ANCOVA	**Non-carriers** (*n*=109)	Volume EMM (mm^3)<br>[95% CI]	291.50<br>[285.48, 297.76]	41.98<br>[40.19, 43.95]	227.01<br>[222.32, 231.61]	726.69<br>[711.54, 740.86]	1071.31<br>[1051.93, 1091.01]	1392.36<br>[1368.03, 1415.29]	1526.84<br>[1502.00, 1550.42]	14.57<br>[14.34, 14.81]	722.20<br>[710.36, 734.60]	17.72<br>[16.83, 18.60]	1680.42<br>[1650.36, 1708.36]	380.45<br>[356.94, 403.57]	326.80<br>[315.32, 338.09]	660.36<br>[644.34, 676.62]	1257.10<br>[1234.77, 1279.52]	1144.63<br>[1123.56, 1166.04]	12335.99<br>[12140.06, 12546.50]
	**Presymptomatic *C9orf72* carriers** (*n*=47)	Volume EMM (mm^3)<br>[95% CI]	290.00<br>[279.81, 299.87]	43.37<br>[40.71, 46.20]	219.33<br>[212.58, 225.70]	713.60<br>[694.53, 732.18]	1031.63<br>[1006.80, 1056.91]	1344.84<br>[1313.10, 1375.93]	1502.04<br>[1467.56, 1535.61]	14.24<br>[13.92, 14.58]	709.50<br>[693.74, 724.70]	17.21<br>[16.00, 18.41]	1591.73<br>[1552.04, 1630.05]	382.39<br>[352.78, 409.79]	330.62<br>[317.37, 343.58]	604.87<br>[577.97, 630.13]	1186.02<br>[1153.61, 1217.64]	1093.17<br>[1066.22, 1121.10]	11914.43<br>[11664.06, 12172.35]
		difference from non-carriers<br>[95% CI]	1.50<br>[-8.77, 11.91]	-1.39<br>[-4.50, 1.69]	7.68<br>[0.33, 15.81]	13.08<br>[-7.97, 34.91]	39.68<br>[15.77, 63.04]	47.52<br>[16.02, 77.90]	24.80<br>[-12.14, 63.41]	0.33<br>[-0.02, 0.65]	12.70<br>[-3.99, 31.50]	0.51<br>[-0.92, 2.02]	88.70<br>[51.17, 125.59]	-1.94<br>[-37.86, 37.04]	-3.82<br>[-21.93, 13.45]	55.50<br>[25.72, 84.94]	71.08<br>[34.04, 106.66]	51.46<br>[21.12, 82.61]	421.56<br>[169.88, 686.26]
		% difference from non-carriers<br>[95% CI]	0.52<br>[-3.01, 4.09]	-3.32<br>[-10.73, 4.03]	3.38<br>[0.15, 6.96]	1.80<br>[-1.10, 4.80]	3.70<br>[1.47, 5.88]	3.41<br>[1.15, 5.59]	1.62<br>[-0.80, 4.15]	2.24<br>[-0.10, 4.44]	1.76<br>[-0.55, 4.36]	2.90<br>[-5.17, 11.42]	5.28<br>[3.04, 7.47]	-0.51<br>[-9.95, 9.74]	-1.17<br>[-6.71, 4.12]	8.40<br>[3.89, 12.86]	5.65<br>[2.71, 8.48]	4.50<br>[1.85, 7.22]	3.42<br>[1.38, 5.56]
		p-value	0.754	0.394	0.049	0.236	**0.004***	**0.002***	0.211	0.065	0.109	0.473	**<0.001***	0.918	0.653	**0.001***	**<0.001***	**0.002***	**0.001***
	**Symptomatic *C9orf72* carriers** (*n*=10)	Volume EMM (mm^3)<br>[95% CI]	239.83<br>[218.15, 258.58]	34.80<br>[28.25, 40.71]	197.55<br>[181.32, 213.82]	615.97<br>[564.77, 668.24]	966.65<br>[914.66, 1022.87]	1288.51<br>[1213.49, 1366.83]	1450.47<br>[1381.74, 1516.14]	13.75<br>[12.83, 14.73]	660.84<br>[616.40, 703.43]	11.76<br>[8.86, 14.52]	1411.95<br>[1338.92, 1485.30]	273.42<br>[193.96, 353.13]	307.59<br>[265.91, 351.63]	511.27<br>[470.67, 552.82]	1110.02<br>[1030.43, 1184.10]	1025.50<br>[967.29, 1086.92]	10917.02<br>[10441.84, 11392.28]
		difference from non-carriers<br>[95% CI]	51.67<br>[34.02, 71.01]	7.18<br>[1.66, 13.01]	29.45<br>[10.53, 46.66]	110.71<br>[66.68, 152.40]	104.67<br>[46.37, 157.76]	103.85<br>[24.64, 175.37]	76.37<br>[2.67, 150.69]	0.82<br>[-0.10, 1.65]	61.35<br>[16.40, 108.69]	5.96<br>[2.50, 9.56]	268.47<br>[186.84, 349.91]	107.02<br>[24.04, 188.08]	19.21<br>[-27.08, 62.45]	149.09<br>[108.31, 190.39]	147.08<br>[81.95, 219.02]	119.13<br>[60.91, 173.76]	1418.97<br>[976.56, 1880.60]
		% difference from non-carriers<br>[95% CI]	17.73<br>[11.67, 24.36]	17.10<br>[3.96, 30.98]	12.98<br>[4.64, 20.55]	15.24<br>[9.18, 20.97]	9.77<br>[4.33, 14.73]	7.46<br>[1.77, 12.59]	5.00<br>[0.17, 9.87]	5.63<br>[-0.68, 11.32]	8.50<br>[2.27, 15.05]	33.65<br>[14.09, 53.93]	15.98<br>[11.12, 20.82]	28.13<br>[6.32, 49.44]	5.88<br>[-8.29, 19.11]	22.58<br>[16.40, 28.83]	11.70<br>[6.52, 17.42]	10.41<br>[5.32, 15.18]	11.50<br>[7.92, 15.24]
		p-value	**<0.001***	**0.021***	**<0.001***	**<0.001***	**<0.001***	**0.002***	0.037	0.065	**0.005***	**<0.001***	**<0.001***	**0.007***	0.379	**<0.001***	**<0.001***	**<0.001***	**<0.001***
GRN ANCOVA	**Non-carriers** (*n*=109)	Volume EMM (mm^3)<br>[95% CI]	291.68<br>[285.68, 297.43]	41.95<br>[40.24, 43.91]	227.30<br>[222.71, 231.83]	726.59<br>[710.98, 741.30]	1073.07<br>[1054.50, 1091.03]	1395.09<br>[1368.56, 1419.53]	1532.97<br>[1507.69, 1557.93]	14.61<br>[14.36, 14.85]	722.01<br>[709.76, 734.93]	17.53<br>[16.66, 18.41]	1678.84<br>[1649.51, 1707.99]	377.71<br>[355.72, 400.15]	326.48<br>[314.62, 337.42]	654.26<br>[638.77, 670.65]	1256.39<br>[1233.98, 1279.17]	1145.24<br>[1124.19, 1166.84]	12337.07<br>[12141.71, 12544.82]
	**Presymptomatic *GRN* carriers** (*n*=57)	Volume EMM (mm^3)<br>[95% CI]	301.06<br>[292.36, 310.58]	41.05<br>[38.32, 43.86]	226.65<br>[220.25, 233.25]	751.04<br>[732.54, 770.01]	1097.22<br>[1076.34, 1118.24]	1416.04<br>[1389.87, 1443.10]	1557.00<br>[1529.34, 1584.33]	14.65<br>[14.36, 14.95]	735.16<br>[719.10, 751.42]	18.80<br>[17.49, 20.29]	1713.47<br>[1680.53, 1748.64]	409.65<br>[379.43, 440.15]	333.81<br>[319.63, 347.51]	658.81<br>[637.20, 680.49]	1279.43<br>[1251.89, 1306.63]	1184.90<br>[1161.07, 1208.64]	12605.84<br>[12380.09, 12834.19]
		difference from non-carriers<br>[95% CI]	-9.38<br>[-18.79, -0.61]	0.90<br>[-2.40, 4.14]	0.65<br>[-6.32, 7.54]	-24.45<br>[-43.40, -6.71]	-24.15<br>[-46.23, -1.79]	-20.96<br>[-50.15, 6.10]	-24.03<br>[-57.69, 9.61]	-0.04<br>[-0.34, 0.24]	-13.15<br>[-29.79, 4.36]	-1.27<br>[-2.78, 0.16]	-34.63<br>[-69.96, 2.77]	-31.94<br>[-68.14, 5.66]	-7.33<br>[-24.81, 9.47]	-4.56<br>[-25.25, 16.09]	-23.05<br>[-52.73, 7.04]	-39.66<br>[-65.47, -13.13]	-268.77<br>[-513.78, -33.42]
		% difference from non-carriers<br>[95% CI]	-3.22<br>[-6.44, -0.21]	2.14<br>[-5.72, 9.88]	0.29<br>[-2.78, 3.32]	-3.36<br>[-5.97, -0.92]	-2.25<br>[-4.31, -0.17]	-1.50<br>[-3.60, 0.44]	-1.57<br>[-3.76, 0.63]	-0.30<br>[-2.34, 1.68]	-1.82<br>[-4.13, 0.60]	-7.26<br>[-15.84, 0.93]	-2.06<br>[-4.17, 0.17]	-8.46<br>[-18.04, 1.50]	-2.25<br>[-7.60, 2.90]	-0.70<br>[-3.86, 2.46]	-1.83<br>[-4.20, 0.56]	-3.46<br>[-5.72, -1.15]	-2.18<br>[-4.16, -0.27]
		p-value	0.053	0.579	0.865	**0.018***	0.040	0.155	0.159	0.778	0.127	0.106	0.060	0.076	0.401	0.678	0.145	**0.002***	0.023
	**Symptomatic *GRN* carriers** (*n*=11)	Volume EMM (mm^3)<br>[95% CI]	240.08<br>[210.08, 264.09]	37.44<br>[31.53, 43.57]	205.51<br>[189.71, 220.85]	649.47<br>[608.95, 690.67]	1020.40<br>[980.72, 1064.16]	1332.62<br>[1283.54, 1382.93]	1433.57<br>[1383.93, 1481.06]	14.06<br>[13.47, 14.60]	666.44<br>[624.12, 706.49]	13.93<br>[10.75, 17.07]	1441.77<br>[1331.38, 1541.93]	425.04<br>[369.05, 482.58]	310.23<br>[274.48, 347.38]	576.61<br>[519.68, 636.26]	1257.76<br>[1217.98, 1307.04]	1140.63<br>[1101.82, 1183.33]	11565.89<br>[11100.92, 12039.38]
		difference from non-carriers<br>[95% CI]	51.59<br>[29.30, 78.58]	4.50<br>[-1.64, 10.61]	21.79<br>[5.57, 38.74]	77.13<br>[35.00, 118.12]	52.66<br>[3.24, 98.35]	62.47<br>[10.07, 117.66]	99.40<br>[49.33, 148.77]	0.55<br>[-0.00, 1.15]	55.57<br>[12.17, 103.12]	3.60<br>[0.26, 6.96]	237.07<br>[130.04, 352.85]	-47.33<br>[-111.76, 16.21]	16.24<br>[-19.25, 51.88]	77.65<br>[18.50, 135.84]	-1.37<br>[-47.51, 37.68]	4.61<br>[-41.71, 48.28]	771.18<br>[356.23, 1189.13]
		% difference from non-carriers<br>[95% CI]	17.69<br>[10.05, 26.94]	10.74<br>[-3.91, 25.28]	9.59<br>[2.45, 17.04]	10.61<br>[4.82, 16.26]	4.91<br>[0.30, 9.17]	4.48<br>[0.72, 8.43]	6.48<br>[3.22, 9.70]	3.76<br>[-0.01, 7.84]	7.70<br>[1.69, 14.28]	20.55<br>[1.51, 39.72]	14.12<br>[7.75, 21.02]	-12.53<br>[-29.59, 4.29]	4.98<br>[-5.90, 15.89]	11.87<br>[2.83, 20.76]	-0.11<br>[-3.78, 3.00]	0.40<br>[-3.64, 4.22]	6.25<br>[2.89, 9.64]
		p-value	**0.001***	0.155	**0.009***	**<0.001***	0.027	**0.015***	**0.001***	0.058	**0.005***	0.027	**<0.001***	0.126	0.347	**0.010***	0.948	0.831	**<0.001***
MAPT ANCOVA	**Non-carriers** (*n*=109)	Volume EMM (mm^3)<br>[95% CI]	291.99<br>[285.93, 298.41]	42.04<br>[40.13, 43.97]	227.48<br>[222.53, 232.84]	727.62<br>[712.01, 743.96]	1073.70<br>[1054.15, 1092.29]	1395.45<br>[1370.08, 1419.54]	1530.96<br>[1505.78, 1556.90]	14.60<br>[14.35, 14.83]	722.76<br>[710.76, 735.51]	17.66<br>[16.80, 18.58]	1682.23<br>[1652.86, 1712.15]	379.80<br>[357.19, 401.59]	326.88<br>[314.96, 338.35]	659.50<br>[643.34, 675.27]	1258.12<br>[1234.63, 1282.02]	1146.16<br>[1124.91, 1168.50]	12352.63<br>[12150.26, 12549.21]
	**Presymptomatic *MAPT* carriers** (*n*=31)	Volume EMM (mm^3)<br>[95% CI]	306.49<br>[295.10, 318.61]	43.82<br>[39.60, 48.02]	231.33<br>[223.90, 238.74]	746.77<br>[721.39, 774.13]	1091.38<br>[1060.17, 1122.06]	1415.80<br>[1376.78, 1454.84]	1555.73<br>[1509.91, 1602.55]	14.78<br>[14.31, 15.25]	740.73<br>[720.77, 760.84]	19.68<br>[17.77, 21.58]	1741.99<br>[1685.63, 1798.13]	399.00<br>[357.64, 439.60]	326.32<br>[302.89, 347.10]	671.62<br>[641.75, 699.12]	1279.37<br>[1247.60, 1312.52]	1168.03<br>[1132.15, 1203.57]	12601.90<br>[12273.70, 12913.22]
		difference from non-carriers<br>[95% CI]	-14.50<br>[-25.96, -4.16]	-1.79<br>[-6.31, 3.07]	-3.85<br>[-12.63, 4.65]	-19.15<br>[-47.40, 7.38]	-17.68<br>[-50.50, 13.41]	-20.35<br>[-63.64, 20.98]	-24.78<br>[-71.78, 21.04]	-0.19<br>[-0.65, 0.30]	-17.97<br>[-39.31, 3.40]	-2.03<br>[-4.27, 0.19]	-59.76<br>[-114.82, -4.97]	-19.20<br>[-63.60, 25.44]	0.56<br>[-23.39, 26.09]	-12.12<br>[-40.56, 16.12]	-21.25<br>[-57.04, 13.44]	-21.87<br>[-57.22, 11.01]	-249.26<br>[-567.80, 59.92]
		% difference from non-carriers<br>[95% CI]	-4.97<br>[-8.89, -1.42]	-4.25<br>[-15.02, 7.30]	-1.69<br>[-5.55, 2.05]	-2.63<br>[-6.51, 1.01]	-1.65<br>[-4.70, 1.25]	-1.46<br>[-4.56, 1.50]	-1.62<br>[-4.69, 1.37]	-1.27<br>[-4.48, 2.03]	-2.49<br>[-5.44, 0.47]	-11.48<br>[-24.19, 1.08]	-3.55<br>[-6.83, -0.30]	-5.06<br>[-16.75, 6.70]	0.17<br>[-7.15, 7.98]	-1.84<br>[-6.15, 2.44]	-1.69<br>[-4.53, 1.07]	-1.91<br>[-4.99, 0.96]	-2.02<br>[-4.60, 0.49]
		p-value	**0.019***	0.437	0.374	0.174	0.262	0.333	0.291	0.455	0.100	0.060	0.033	0.401	0.969	0.409	0.241	0.237	0.116
	**Symptomatic *MAPT* carriers** (*n*=12)	Volume EMM (mm^3)<br>[95% CI]	263.19<br>[242.16, 281.62]	31.50<br>[25.21, 37.76]	194.89<br>[178.57, 211.98]	689.59<br>[648.12, 726.27]	1032.59<br>[989.20, 1073.68]	1324.24<br>[1273.75, 1368.50]	1457.42<br>[1391.99, 1514.01]	13.68<br>[13.02, 14.32]	663.32<br>[630.65, 696.13]	14.66<br>[12.06, 17.33]	1523.96<br>[1442.04, 1604.07]	405.39<br>[337.72, 469.02]	306.76<br>[280.02, 333.38]	526.80<br>[478.20, 575.09]	1222.93<br>[1164.11, 1280.03]	1139.22<br>[1089.93, 1186.37]	11564.68<br>[11073.85, 12015.54]
		difference from non-carriers<br>[95% CI]	28.79<br>[9.97, 50.00]	10.54<br>[4.27, 17.16]	32.59<br>[16.75, 48.60]	38.03<br>[-1.91, 81.75]	41.11<br>[0.09, 84.02]	71.21<br>[26.57, 120.48]	73.54<br>[19.01, 133.71]	0.92<br>[0.28, 1.54]	59.44<br>[27.56, 91.91]	2.99<br>[0.19, 5.91]	158.27<br>[63.14, 244.94]	-25.59<br>[-90.58, 39.85]	20.12<br>[-6.78, 46.89]	132.70<br>[84.71, 183.07]	35.19<br>[-26.08, 101.44]	6.95<br>[-43.03, 59.45]	787.95<br>[276.62, 1304.45]
		% difference from non-carriers<br>[95% CI]	9.86<br>[3.41, 17.12]	25.07<br>[10.15, 40.82]	14.33<br>[7.36, 21.36]	5.23<br>[-0.26, 11.24]	3.83<br>[0.01, 7.83]	5.10<br>[1.90, 8.63]	4.80<br>[1.24, 8.73]	6.28<br>[1.91, 10.53]	8.22<br>[3.81, 12.72]	16.96<br>[1.05, 33.47]	9.41<br>[3.75, 14.56]	-6.74<br>[-23.85, 10.49]	6.15<br>[-2.08, 14.34]	20.12<br>[12.84, 27.76]	2.80<br>[-2.07, 8.06]	0.61<br>[-3.75, 5.19]	6.38<br>[2.24, 10.56]
		p-value	**0.003***	**0.001***	**<0.001***	0.061	0.051	**0.003***	**0.013***	**0.002***	**<0.001***	0.034	**0.001***	0.430	0.146	**<0.001***	0.238	0.787	**0.001***

Supplementary Table 2. Thalamic subregional mean diffusivity comparisons between mutation carriers and non-carriers. Negative differences indicate higher (right and left averaged) MD values in mutation carriers relative to non-carriers. Uncorrected *p*-values are shown; asterisked bold *p*-values indicate significance at $p < 0.05$ after correction for multiple comparisons. The covariates included were age and sex. The 95% confidence interval limits for the percentage differences were calculated from the confidence interval limits for the raw group differences in estimated marginal means.

ANCOVA – univariate analysis of covariance, CI – confidence interval, EMM – estimated marginal mean from ANCOVA, MD – mean diffusivity. Thalamic subregions: AV – anteroventral, LD – laterodorsal, LGN – lateral geniculate, LP – lateral posterior, MGN – medial geniculate, PuMl – lateral part of the medial pulvinar, PuMm – medial part of the medial pulvinar, VAM – ventral anterior and ventromedial, VLa – ventral lateral anterior, VLp – ventral lateral posterior, VPL – ventral posterolateral.

Group			AV	LD	LP	VAM	VLa	VLp	VPL	Intralaminar	Medial	LGN	MGN	PuMm	PuMl	Non-medial pulvinar	Whole thalamus
C9orf72<br>ANCOVA	**Non-carriers**<br>(*n*=109)	MD EMM (x 10^{-3} mm^2/s)<br>[95% CI]	0.896<br>[0.884, 0.907]	1.095<br>[1.078, 1.111]	0.978<br>[0.966, 0.992]	0.753<br>[0.748, 0.759]	0.731<br>[0.727, 0.735]	0.737<br>[0.733, 0.741]	0.703<br>[0.699, 0.707]	0.723<br>[0.718, 0.729]	0.793<br>[0.787, 0.799]	0.730<br>[0.723, 0.737]	0.768<br>[0.761, 0.776]	0.858<br>[0.845, 0.870]	0.871<br>[0.862, 0.880]	0.752<br>[0.747, 0.757]	0.774<br>[0.769, 0.780]
	**Presymptomatic *C9orf72* carriers**<br>(*n*=47)	MD EMM (x 10^{-3} mm^2/s)<br>[95% CI]	0.932<br>[0.911, 0.954]	1.120<br>[1.099, 1.145]	0.992<br>[0.975, 1.010]	0.771<br>[0.760, 0.781]	0.737<br>[0.729, 0.745]	0.741<br>[0.733, 0.748]	0.703<br>[0.696, 0.711]	0.725<br>[0.717, 0.733]	0.803<br>[0.794, 0.813]	0.737<br>[0.724, 0.749]	0.777<br>[0.765, 0.788]	0.901<br>[0.880, 0.921]	0.912<br>[0.899, 0.927]	0.769<br>[0.760, 0.777]	0.787<br>[0.778, 0.795]
		difference from non-carriers<br>[95% CI]	-0.036<br>[-0.062, -0.012]	-0.026<br>[-0.055, -0.002]	-0.013<br>[-0.035, 0.007]	-0.017<br>[-0.028, -0.007]	-0.006<br>[-0.014, 0.002]	-0.004<br>[-0.013, 0.005]	-0.001<br>[-0.009, 0.007]	-0.002<br>[-0.011, 0.008]	-0.010<br>[-0.021, 0.001]	-0.007<br>[-0.020, 0.006]	-0.009<br>[-0.022, 0.005]	-0.043<br>[-0.065, -0.021]	-0.041<br>[-0.057, -0.026]	-0.016<br>[-0.025, -0.007]	-0.012<br>[-0.021, -0.003]
		% difference from non-carriers<br>[95% CI]	-4.040<br>[-6.898, -1.343]	-2.364<br>[-5.019, -0.165]	-1.349<br>[-3.565, 0.709]	-2.316<br>[-3.779, -0.875]	-0.823<br>[-1.966, 0.253]	-0.511<br>[-1.703, 0.621]	-0.098<br>[-1.210, 1.028]	-0.238<br>[-1.466, 1.109]	-1.295<br>[-2.681, 0.187]	-0.902<br>[-2.771, 0.866]	-1.149<br>[-2.960, 0.658]	-5.062<br>[-7.632, -2.468]	-4.721<br>[-6.513, -3.017]	-2.148<br>[-3.336, -0.906]	-1.563<br>[-2.764, -0.382]
		p-value	**0.004***	0.062	0.213	**0.002***	0.152	0.368	0.874	0.692	0.075	0.341	0.195	**0.001***	**<0.001***	**0.001***	**0.010***
	**Symptomatic *C9orf72* carriers**<br>(*n*=10)	MD EMM (x 10^{-3} mm^2/s)<br>[95% CI]	1.011<br>[0.965, 1.060]	1.168<br>[1.113, 1.225]	1.041<br>[0.999, 1.081]	0.798<br>[0.773, 0.824]	0.754<br>[0.736, 0.772]	0.761<br>[0.744, 0.779]	0.715<br>[0.701, 0.729]	0.747<br>[0.726, 0.771]	0.852<br>[0.823, 0.881]	0.769<br>[0.724, 0.814]	0.796<br>[0.764, 0.827]	1.040<br>[0.966, 1.118]	1.021<br>[0.971, 1.072]	0.794<br>[0.771, 0.817]	0.824<br>[0.803, 0.846]
		difference from non-carriers<br>[95% CI]	-0.115<br>[-0.159, -0.071]	-0.074<br>[-0.139, -0.013]	-0.063<br>[-0.104, -0.021]	-0.044<br>[-0.072, -0.020]	-0.023<br>[-0.043, -0.003]	-0.024<br>[-0.041, -0.007]	-0.013<br>[-0.028, 0.003]	-0.024<br>[-0.045, -0.003]	-0.059<br>[-0.090, -0.029]	-0.039<br>[-0.086, 0.008]	-0.028<br>[-0.060, 0.005]	-0.182<br>[-0.265, -0.104]	-0.150<br>[-0.205, -0.097]	-0.042<br>[-0.066, -0.018]	-0.050<br>[-0.076, -0.024]
		% difference from non-carriers<br>[95% CI]	-12.836<br>[-17.776, -7.933]	-6.741<br>[-12.729, -1.147]	-6.420<br>[-10.637, -2.110]	-5.901<br>[-9.557, -2.627]	-3.205<br>[-5.896, -0.429]	-3.307<br>[-5.577, -0.935]	-1.799<br>[-3.970, 0.466]	-3.268<br>[-6.223, -0.470]	-7.50<br>[-11.354, -3.629]	-5.287<br>[-11.821, 1.034]	-3.605<br>[-7.867, 0.678]	-21.227<br>[-30.844, -12.118]	-17.234<br>[-23.472, -11.134]	-5.518<br>[-8.721, -2.444]	-6.414<br>[-9.755, -3.141]
		p-value	**<0.001***	**0.014***	**0.002***	**0.001***	**0.013***	**0.004***	0.097	0.028	**<0.001***	0.104	0.076	**<0.001***	**<0.001***	**0.001***	**<0.001***
GRN<br>ANCOVA	**Non-carriers**<br>(*n*=109)	MD EMM (x 10^{-3} mm^2/s)<br>[95% CI]	0.900<br>[0.888, 0.912]	1.102<br>[1.085, 1.119]	0.982<br>[0.969, 0.995]	0.755<br>[0.749, 0.760]	0.731<br>[0.727, 0.736]	0.737<br>[0.733, 0.742]	0.703<br>[0.699, 0.707]	0.724<br>[0.719, 0.729]	0.794<br>[0.788, 0.801]	0.731<br>[0.723, 0.739]	0.769<br>[0.761, 0.776]	0.861<br>[0.850, 0.874]	0.874<br>[0.865, 0.884]	0.753<br>[0.748, 0.758]	0.776<br>[0.770, 0.781]
	**Presymptomatic *GRN* carriers**<br>(*n*=57)	MD EMM (x 10^{-3} mm^2/s)<br>[95% CI]	0.916<br>[0.901, 0.932]	1.121<br>[1.098, 1.145]	0.985<br>[0.971, 0.999]	0.765<br>[0.757, 0.771]	0.736<br>[0.730, 0.742]	0.741<br>[0.735, 0.747]	0.706<br>[0.700, 0.712]	0.726<br>[0.720, 0.732]	0.800<br>[0.792, 0.808]	0.737<br>[0.730, 0.746]	0.773<br>[0.764, 0.783]	0.863<br>[0.849, 0.879]	0.877<br>[0.865, 0.889]	0.756<br>[0.750, 0.763]	0.780<br>[0.774, 0.786]
		difference from non-carriers<br>[95% CI]	-0.016<br>[-0.036, 0.004]	-0.019<br>[-0.047, 0.007]	-0.003<br>[-0.022, 0.015]	-0.010<br>[-0.019, -0.001]	-0.005<br>[-0.012, 0.002]	-0.004<br>[-0.011, 0.003]	-0.003<br>[-0.009, 0.004]	-0.002<br>[-0.010, 0.006]	-0.006<br>[-0.017, 0.005]	-0.006<br>[-0.017, 0.005]	-0.005<br>[-0.016, 0.006]	-0.002<br>[-0.021, 0.016]	-0.002<br>[-0.017, 0.012]	-0.003<br>[-0.011, 0.005]	-0.004<br>[-0.012, 0.004]
		% difference from non-carriers<br>[95% CI]	-1.807<br>[-4.014, 0.489]	-1.739<br>[-4.268, 0.616]	-0.287<br>[-2.211, 1.519]	-1.320<br>[-2.540, -0.096]	-0.624<br>[-1.617, 0.330]	-0.496<br>[-1.488, 0.453]	-0.407<br>[-1.336, 0.495]	-0.273<br>[-1.359, 0.785]	-0.768<br>[-2.079, 0.575]	-0.792<br>[-2.267, 0.620]	-0.608<br>[-2.074, 0.815]	-0.244<br>[-2.448, 1.911]	-0.276<br>[-1.964, 1.337]	-0.425<br>[-1.456, 0.615]	-0.508<br>[-1.498, 0.539]
		p-value	0.099	0.176	0.764	0.031	0.213	0.334	0.392	0.621	0.268	0.345	0.448	0.828	0.755	0.430	0.321
	**Symptomatic *GRN* carriers**<br>(*n*=11)	MD EMM (x 10^{-3} mm^2/s)<br>[95% CI]	1.053<br>[0.986, 1.127]	1.244<br>[1.195, 1.295]	1.074<br>[1.035, 1.114]	0.813<br>[0.769, 0.862]	0.772<br>[0.746, 0.801]	0.778<br>[0.753, 0.803]	0.723<br>[0.698, 0.750]	0.764<br>[0.735, 0.796]	0.886<br>[0.838, 0.935]	0.771<br>[0.733, 0.817]	0.809<br>[0.777, 0.844]	1.003<br>[0.917, 1.101]	0.949<br>[0.903, 0.999]	0.801<br>[0.762, 0.845]	0.833<br>[0.802, 0.867]
		difference from non-carriers<br>[95% CI]	-0.153<br>[-0.238, -0.077]	-0.143<br>[-0.194, -0.093]	-0.091<br>[-0.132, -0.051]	-0.059<br>[-0.105, -0.016]	-0.041<br>[-0.072, -0.012]	-0.040<br>[-0.067, -0.014]	-0.020<br>[-0.050, 0.007]	-0.040<br>[-0.074, -0.009]	-0.092<br>[-0.142, -0.044]	-0.041<br>[-0.085, -0.002]	-0.041<br>[-0.082, -0.003]	-0.142<br>[-0.235, -0.058]	-0.075<br>[-0.123, -0.028]	-0.048<br>[-0.092, -0.010]	-0.057<br>[-0.096, -0.023]
		% difference from non-carriers<br>[95% CI]	-17.017<br>[-26.501, -8.542]	-12.945<br>[-17.635, -8.480]	-9.300<br>[-13.459, -5.239]	-7.806<br>[-13.902, -2.077]	-5.590<br>[-9.806, -1.601]	-5.452<br>[-9.129, -1.904]	-2.905<br>[-7.090, 1.044]	-5.553<br>[-10.159, -1.217]	-11.539<br>[-17.829, -5.521]	-5.554<br>[-11.618, -0.339]	-5.311<br>[-10.620, -0.450]	-16.495<br>[-27.315, -6.696]	-8.543<br>[-14.123, -3.256]	-6.327<br>[-12.151, -1.343]	-7.343<br>[-12.363, -2.946]
		p-value	**0.001***	**<0.001***	**<0.001***	**0.015***	**0.007***	**0.002***	0.139	**0.010***	**0.002***	0.054	0.029	**0.002***	**0.002***	**0.022***	**0.001***
MAPT<br>ANCOVA	**Non-carriers**<br>(*n*=109)	MD EMM (x 10^{-3} mm^2/s)<br>[95% CI]	0.897<br>[0.886, 0.909]	1.096<br>[1.079, 1.114]	0.980<br>[0.965, 0.992]	0.754<br>[0.748, 0.759]	0.731<br>[0.727, 0.735]	0.737<br>[0.733, 0.741]	0.703<br>[0.699, 0.707]	0.724<br>[0.719, 0.729]	0.793<br>[0.787, 0.799]	0.730<br>[0.723, 0.738]	0.768<br>[0.761, 0.776]	0.859<br>[0.847, 0.871]	0.872<br>[0.863, 0.881]	0.753<br>[0.748, 0.758]	0.775<br>[0.770, 0.780]
	**Presymptomatic *MAPT* carriers**<br>(*n*=31)	MD EMM (x 10^{-3} mm^2/s)<br>[95% CI]	0.899<br>[0.875, 0.925]	1.119<br>[1.088, 1.151]	0.992<br>[0.967, 1.016]	0.746<br>[0.736, 0.756]	0.723<br>[0.714, 0.732]	0.729<br>[0.719, 0.739]	0.694<br>[0.685, 0.703]	0.714<br>[0.704, 0.724]	0.787<br>[0.774, 0.799]	0.731<br>[0.715, 0.747]	0.762<br>[0.747, 0.779]	0.853<br>[0.835, 0.870]	0.873<br>[0.856, 0.893]	0.751<br>[0.740, 0.762]	0.770<br>[0.759, 0.780]
		difference from non-carriers<br>[95% CI]	-0.002<br>[-0.028, 0.023]	-0.023<br>[-0.056, 0.010]	-0.012<br>[-0.040, 0.015]	0.007<br>[-0.005, 0.019]	0.008<br>[-0.002, 0.018]	0.008<br>[-0.002, 0.018]	0.008<br>[-0.001, 0.020]	0.009<br>[-0.003, 0.023]	0.006<br>[-0.008, 0.020]	-0.000<br>[-0.017, 0.016]	0.007<br>[-0.012, 0.023]	0.006<br>[-0.013, 0.024]	-0.001<br>[-0.021, 0.018]	0.002<br>[-0.008, 0.012]	0.005<br>[-0.006, 0.016]
		% difference from non-carriers<br>[95% CI]	-0.189<br>[-3.095, 2.611]	-2.067<br>[-5.135, 0.890]	-1.230<br>[-4.059, 1.566]	0.951<br>[-0.656, 2.476]	1.135<br>[-0.233, 2.469]	1.135<br>[-0.308, 2.486]	1.203<br>[-0.206, 2.802]	1.292<br>[-0.417, 3.122]	0.802<br>[-0.980, 2.485]	-0.032<br>[-2.366, 2.129]	0.889<br>[-1.589, 2.944]	0.676<br>[-1.486, 2.849]	-0.142<br>[-2.451, 2.034]	0.215<br>[-1.114, 1.638]	0.661<br>[-0.753, 2.061]
		p-value	0.893	0.171	0.377	0.193	0.107	0.119	0.102	0.125	0.359	0.978	0.446	0.553	0.897	0.779	0.366
	**Symptomatic *MAPT* carriers**<br>(*n*=12)	MD EMM (x 10^{-3} mm^2/s)<br>[95% CI]	1.032<br>[0.991, 1.079]	1.244<br>[1.190, 1.301]	1.059<br>[1.017, 1.102]	0.802<br>[0.782, 0.823]	0.769<br>[0.756, 0.784]	0.771<br>[0.758, 0.788]	0.726<br>[0.712, 0.743]	0.758<br>[0.739, 0.783]	0.856<br>[0.829, 0.886]	0.755<br>[0.720, 0.793]	0.816<br>[0.789, 0.845]	1.058<br>[0.991, 1.130]	0.936<br>[0.906, 0.975]	0.791<br>[0.771, 0.812]	0.823<br>[0.804, 0.846]
		difference from non-carriers<br>[95% CI]	-0.135<br>[-0.186, -0.091]	-0.147<br>[-0.201, -0.096]	-0.080<br>[-0.127, -0.036]	-0.048<br>[-0.072, -0.026]	-0.038<br>[-0.054, -0.024]	-0.034<br>[-0.053, -0.019]	-0.023<br>[-0.042, -0.007]	-0.035<br>[-0.061, -0.013]	-0.063<br>[-0.093, -0.037]	-0.024<br>[-0.062, 0.009]	-0.047<br>[-0.081, -0.017]	-0.199<br>[-0.274, -0.135]	-0.064<br>[-0.101, -0.033]	-0.038<br>[-0.061, -0.017]	-0.049<br>[-0.074, -0.027]
		% difference from non-carriers<br>[95% CI]	-15.083<br>[-20.761, -10.154]	-13.438<br>[-18.366, -8.742]	-8.159<br>[-12.977, -3.672]	-6.403<br>[-9.616, -3.472]	-5.181<br>[-7.401, -3.252]	-4.661<br>[-7.132, -2.585]	-3.258<br>[-5.960, -1.056]	-4.792<br>[-8.420, -1.853]	-7.945<br>[-11.700, -4.602]	-3.335<br>[-8.542, 1.168]	-6.129<br>[-10.519, -2.245]	-23.171<br>[-31.909, -15.694]	-7.356<br>[-11.626, -3.819]	-5.069<br>[-8.151, -2.273]	-6.270<br>[-9.498, -3.504]
		p-value	**<0.001***	**<0.001***	**<0.001***	**<0.001***	**<0.001***	**0.001***	**0.004***	**0.003***	**<0.001***	0.189	**0.003***	**<0.001***	**<0.001***	**0.001***	**<0.001***

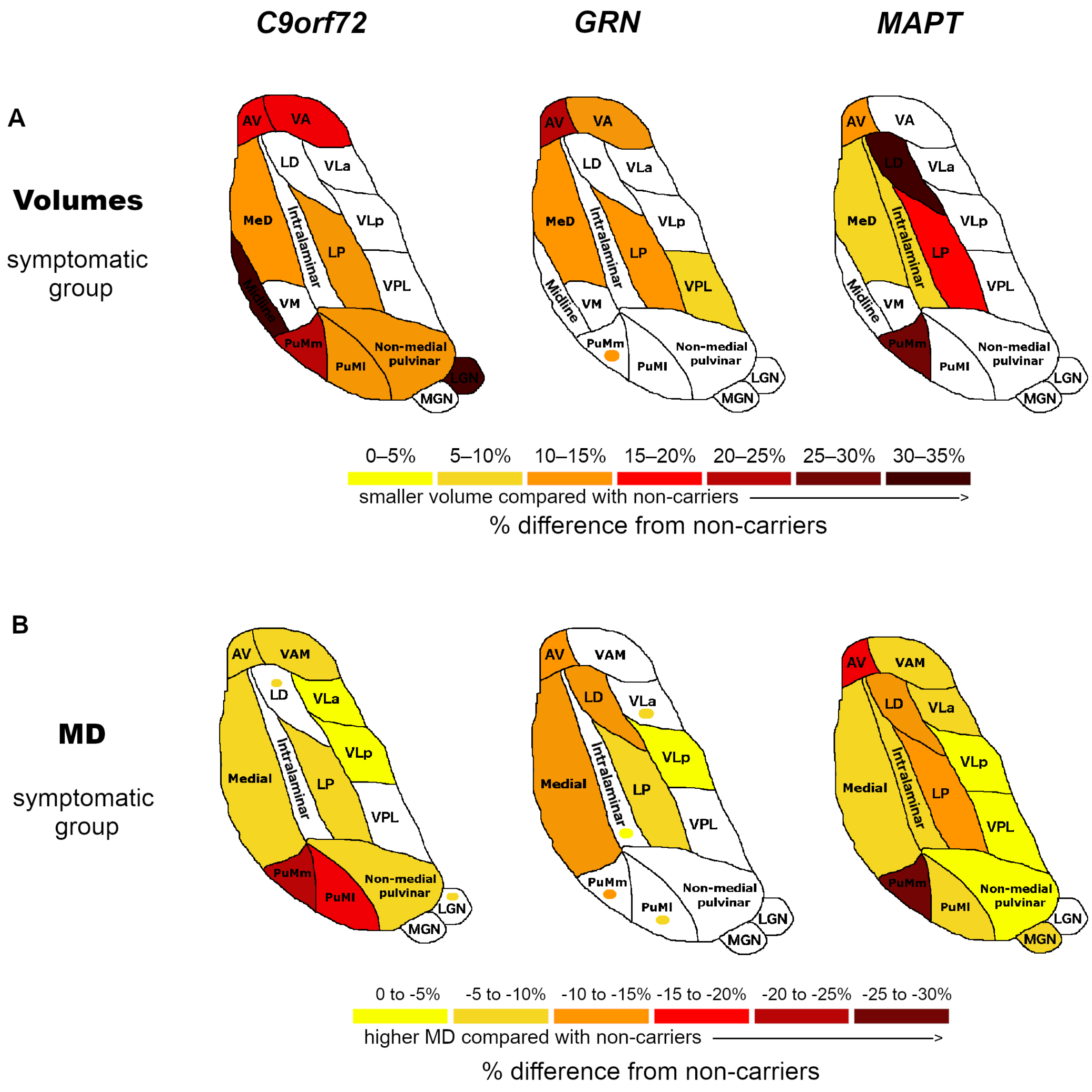

Supplementary Figure 1. Thalamic subregional volumetric (panel A) and mean diffusivity (MD) (panel B) differences in symptomatic mutation carriers (10 *C9orf72*, 11 *GRN*, 9 *MAPT*) compared with 28 mutation non-carriers over the age of 52 years.

Panel A. Positive differences indicate smaller (right and left summed) volumes in mutation carriers relative to non-carriers on univariate analyses of covariance (ANCOVAs) with age, sex and total intracranial volume as covariates, followed by pairwise comparisons. Panel B. Negative differences indicate higher (right and left averaged) MD values in mutation carriers relative to non-carriers on univariate analyses of covariance (ANCOVAs) with age and sex as covariates, followed by pairwise comparisons. The volume and MD comparisons with non-carriers were carried out using separate analyses of covariance (ANCOVAs) for each subregion and genetic group for consistency with the main analyses.

Differences for all fully coloured subregions in panels A and B are significant at $p < 0.05$ after correction for multiple comparisons using the Benjamini-Hochberg method (carried out separately for volume and MD comparisons for each genetic group). Differences for subregions annotated with an oval border significance (corrected $p = 0.05$–0.06).

MD – mean diffusivity. Thalamic subregions: AV – anteroventral, LD – laterodorsal, LGN – lateral geniculate, LP – lateral posterior, MeD – mediodorsal, MGN – medial geniculate, PuMl – lateral part of the medial pulvinar, PuMm – medial part of the medial pulvinar, VA – ventral anterior, VAM – ventral anterior and ventromedial, VLa – ventral lateral anterior, VLp – ventral lateral posterior, VM – ventromedial, VPL – ventral posterolateral.
